# A new multiplex qPCR assay to detect and differentiate big cat species in the illegal wildlife trade

**DOI:** 10.1038/s41598-023-36776-z

**Published:** 2023-06-16

**Authors:** Carol S. Henger, Dyan J. Straughan, Charles C. Y. Xu, Batya R. Nightingale, Heidi E. Kretser, Mary K. Burnham-Curtis, Denise McAloose, Tracie A. Seimon

**Affiliations:** 1 Zoological Health Program, Wildlife Conservation Society, Bronx, NY USA; 2 OLE-National Fish and Wildlife Forensic Laboratory, United States Fish and Wildlife Service, Ashland, OR USA; 3 Redpath Museum and Department of Biology, McGill University, Montreal, QC Canada; 4 Global Conservation Program, Wildlife Conservation Society, Bronx, NY USA; 5 Department of Natural Resources and the Environment, Cornell University, Ithaca, NY USA

**Keywords:** Genetics, Molecular biology

## Abstract

All species of big cats, including tigers, cheetahs, leopards, lions, snow leopards, and jaguars, are protected under the Convention on the International Trade in Endangered Species (CITES). This is due in large part to population declines resulting from anthropogenic factors, especially poaching and the unregulated and illegal trade in pelts, bones, teeth and other products that are derived from these iconic species. To enhance and scale up monitoring for big cat products in this trade, we created a rapid multiplex qPCR test that can identify and differentiate DNA from tiger (*Panthera tigris*), cheetah (*Acinonyx jubatus*), leopard (*Panthera pardus*), lion (*Panthera leo*), snow leopard (*Panthera uncia*), and jaguar (*Panthera onca*) in wildlife products using melt curve analysis to identify each species by its unique melt peak temperature. Our results showed high PCR efficiency (> 90%), sensitivity (detection limit of 5 copies of DNA per PCR reaction) and specificity (no cross amplification between each of the 6 big cat species). When paired with a rapid (< 1 h) DNA extraction protocol that amplifies DNA from bone, teeth, and preserved skin, total test time is less than three hours. This test can be used as a screening method to improve our understanding of the scale and scope of the illegal trade in big cats and aid in the enforcement of international regulations that govern the trade in wildlife and wildlife products, both ultimately benefiting the conservation of these species worldwide.

## Introduction

Big cats are among the most iconic and culturally and spiritually significant animals on the planet^1^. As apex predators, they are also important in maintaining biodiversity and ecosystem function^2^. An estimated 100,000 wild tigers (*Panthera tigris*) ranged across Asia 100 years ago^3^. However, current estimates suggest a global tiger population of between 3,726 and 5,578 individuals^4^. This decline is due to anthropogenic factors, including poaching to supply an increasing demand for tiger parts and products through the illegal wildlife trade^5^. As an example, there were 2,205 confiscations globally of tigers and their parts between January 2000 and June 2022^6^. The illegal wildlife trade is a multi-billion-dollar industry^7^ and within this trade, tiger parts and products are among the most profitable and broadly used^8,9^. All parts of the tiger are valued. Tiger bones are used in a variety of traditional medicines, tonics, wine, and plasters. Skin, whiskers, and teeth are used as ornaments and charms, and skins and carcasses are used as household decorations and trophies^10–12^. Much of the tiger trade is driven by demand from Asia, where tigers symbolize strength, power, and status, and where tiger parts have been used in traditional Chinese medicine (TCM) for hundreds of years^13,14^.

 The international trade of tigers or tiger parts is illegal^15^. Additional regional restrictions exist and in 1993 China enacted a domestic trade ban on tiger parts, including those used as ingredients in traditional medicines^16^. In response to these restrictions and declining tiger populations, some suppliers have begun to substitute products from other species for tiger products^5^. For example, the international trade of products from captive African lions (*Panthera leo*) is legal under CITES (Convention on the International Trade in Endangered Species^15^, so lion bones have been used as a substitute for tiger bones in the production of medicinal wine^17^. This has corresponded to an increase in annual export permits for lion skeletons from South Africa and Namibia to South Asia from an average of 314 skeletons/year between 2008 and 2011 to an average of 1,312/year between 2013 to 2015. More than 3,400 skeletons were exported between 2014 and 2016^18^. Overall, an estimated 6,000 skeletons weighing more than 70 tonnes have been exported to South Asia since 2008^18^. Leopard bones are also used as substitutes for tiger bones for health tonics and TCM^19^. Over 5,000 skins, carcasses, stuffed, and live leopards (*Panthera pardus*) have been seized from the illegal trade in Asia since 2000^19^. Snow leopards (*Panthera uncia*) in Central Asia are also poached for their skins and to serve as tiger bone substitutes^20,21^. Cheetahs (*Acinonyx jabatus*) from Africa and Asia are primarily trafficked as pets and sold for their skin, teeth, and other parts^22,23^. The illegal trade of big cat species is not restricted to Africa and Asia, as harvesting of jaguars (*Panthera onca*) from Latin America is increasing^12^. Between August 2014 and February 2015, 186 jaguar canine teeth, representing up to 100 individuals that were enroute to China were seized by Bolivian authorities^24^. Jaguar paste, a glue-like material made by boiling down jaguar carcasses, is being used in China^25^ as a substitute for tiger bone glue/paste^26^. Online markets provide a platform that enables the illegal trade of jaguar parts from Latin America to China^27^. The lack of sufficient resources for adequate monitoring and forensic analysis of products confiscated along the wildlife trafficking supply chain or at international destinations prevents us from understanding the true scope and scale of the illegal trade in big cats, which has the potential to be much greater than current estimates.

To reduce wildlife trafficking of big cats and reverse population declines, a better understanding of the scale of the global trade and improved enforcement of CITES regulations is imperative^28^. Much of the illegal trade of big cats involves bones, teeth, and other body parts^9^ that can be difficult to identify to species using visual inspection alone. The most sensitive method for species identification is genetic analysis and testing often requires sending big cat products to specialized laboratories that can run forensic tests such as capillary electrophoresis^29^, genetic sequencing^30^, and metabarcoding^31^. The process is often time-consuming and expensive, may be restricted to the highest priority or urgent cases which limits the number of products that can be tested, and can result in long delays for prosecution if appropriate laboratory facilities and protocols for performing these types of advanced analyses are not locally or regionally available. An easy-to-use screening tool that (1) could quickly identify a species in suspected wildlife products, (2) could be used for on-site testing at local or regional checkpoints, and (3) is scalable, would bridge the gap between current low volume and capacity testing to more comprehensive monitoring and forensic analysis, and provide a better understanding of the scope and scale of big cat products entering the trade. Furthermore, the tool would increase the specimen/sample throughput, aiding in enforcement of CITES regulations.

Melt curve analysis measures the fluorescence produced by DNA intercalating dyes, such as SYBR Green, at different temperatures to produce a melt peak, the temperature at which the two strands of a DNA amplicon (produced from a PCR reaction) dissociate^32^. Thus, melt peaks can serve as proxies to differentiate DNA amplicons based on their sequence identities. Melt curve analysis has been well-established within wildlife forensics and has been used to identify illegal bushmeat^33^, detect threatened shark species^34^, and distinguish African from Asian ivory^35^. Melt curve qPCR analysis is fast and does not require purchase of expensive qPCR probes and there are no downstream processes typical of standard PCR methods such as DNA sequencing or gel electrophoresis. In addition, melt curve analysis is a standard component of many software programs that are available on modern qPCR machines.

Here we describe the development of a user-friendly multiplex qPCR DNA screening test that utilizes melt curve analysis to identify and differentiate six big cat species: tiger (*P. tigris*), lion (*P. leo*), leopard (*P. pardus*), jaguar (*P. onca*), snow leopard (*P. uncia*), and cheetah (*A. jubatus*). Three laboratories pilot tested the DNA test for PCR efficiency, sensitivity, and specificity to the target big cat species. Test development and extraction methodologies were performed on a number of different tissue and sample types, including teeth, unprocessed bone, and bone steeped in alcohol. This quick yet sensitive test will help to expand our understanding of the scale and the scope of illegal wildlife trafficking and trade of big cats.

## Methods

### Samples used for testing

Tissue and bone samples were collected during routine necropsy procedures at the Wildlife Conservation Society (WCS), Bronx, NY. Permission was obtained for all samples. Additionally, the United States Fish and Wildlife Service (USFWS) Forensic Laboratory, Ashland, OR provided tissue and bone samples from their reference collections. Species identification of all samples had been previously confirmed. The latter were obtained opportunistically or through contributions of samples from known species (see Supplementary Information for details). An ocelot sample was generously donated by the American Museum of Natural History (AMNH).

A mock tiger bone wine sample was prepared using archived tiger bone (postmortem toe sample approximately 40 cm^3^) provided by the Conservation and Research Department of the Granby Zoo, Quebec, Canada. The tiger bone was steeped in 50 mL of commercially available baiju (小黄鹤楼白酒; Little Yellow Crane White Liquor made from neutral spirits, glutinous rice, sorghum, wheat, rice, and corn) and stored at ambient temperature. Bone samples were archived at ambient temperatures for up to 31 years before DNA extraction. Soft tissue samples were archived at both institutions at − 80 °C for up to 32 years prior to DNA extraction. Formalin-fixed paraffin-embedded (FFPE**)** samples were stored at ambient temperature and extracted from 6 to 41 years after embedding. Fecal samples were opportunistically collected (Bronx Zoo) and DNA extraction was performed within four hours of sample collection.

All tissue, blood, FFPE, bone, mock tiger bone wine, and fecal samples used in the experiments are listed in Table 1. No live animals were sacrificed or euthanized for this study. All samples were either collected from archived material collected opportunistically from animals that died naturally or were humanely euthanized because of severe illness due to natural disease processes in a zoological collection, archived bone material found in a museum collection, or products confiscated and archived from the wildlife trade. All experiments were performed in accordance with all relevant guidelines and regulations. Institutional Care and Use Committee (IACUC) approval for this project was not required as WCS institutional requirements for IACUC review do not include standard veterinary care that take place within the zoological facilities. Sampling, capture and release of captive animals for standard veterinary care, and or sampling after euthanasia are all standard techniques and procedures for clinical and pathology examinations or investigations and are in compliance with the Association for Zoos and Aquaria (AZA). The welfare of animals that provided archived samples included in this study was considered throughout their care, with analgesics used as deemed necessary and methods of euthanasia accepted by the Association for Zoos and Aquaria (AZA) employed. In addition, all experiments are in accordance with ARRIVE guidelines (arriveguidelines.org).

**Table 1:** All tissue, blood, FFPE, bone, mock tiger bone wine, and fecal samples used in the experiments

Scientific name	Common name	No. of Samples (WCS/USFWS)	Sample Type (WCS/USFWS)
***Acinonyx jubatus***	Cheetah	0/10	NA^†^/ liver, muscle, bone
***Acinonyx jubatus jubatus***	Southern African Cheetah	1/6	Liver/ blood
*Bos taurus*	Cow	0/3	NA^†^/ blood
*Canis familiaris*	Domestic Dog	1/0	Cheek swab/ NA^†^
*Caracal caracal*	Caracal	0/4	NA^†^/ blood
*Equus caballus*	Domestic Horse	1/0	Bone/ NA^†^
*Felis catus*	Domestic Cat	1/0	Liver/ NA^†^
*Felis nigripes*	Black-footed cat	0/8	NA^†^/ blood
*Felis silvestris*	European Wild Cat	1/0	Liver (FFPE*)/ NA^†^
*Homo sapiens*	Human	1/0	Cheek swab/ NA^†^
*Leopardus pardalis*	Ocelot	1/0	Muscle/ NA^†^
*Lynx rufus*	Bobcat	1/0	Muscle/ NA^†^
*Lynx canadensis*	Canada Lynx	1/0	Liver/ NA^†^
*Neofelis nebulosa*	Clouded Leopard	1/0	Feces/ NA^†^
*Otocolobus manual*	Pallas’s Cat	1/3	Liver/ tongue, blood
*Ovis dalli*	Dall sheep	0/3	NA^†^/ blood
*Ovis vignei*	Urial	0/2	NA^†^/ blood
***Panthera leo***	Lion	3/11	Feces/ muscle, skin, blood
***Panthera leo krugeri***	Transvaal lion	4/2	Kidney,feces/ blood
***Panthera onca***	Jaguar	1/12	Liver (FFPE*)/ muscle, skin, liver, bone
***Panthera onca Goldmani***	Goldman’s Jaguar	0/1	NA^†^/ tissue
***Panthera pardus***	Leopard	2/13	Spleen, bone/ muscle, blood, skin
***Panthera pardus orientalis***	Amur Leopard	1/3	Liver (FFPE*)/ muscle, blood
***Panthera pardus sindica***	Sind leopard	0/1	NA^†^/ muscle
***Panthera tigris***	Tiger	0/4	NA^†^/ muscle, bone
***Panthera tigris altaica***	Amur Tiger	13/13	Kidney, liver, liver (FFPE*), bone, feces/ liver, muscle
***Panthera tigris jacksoni***	Malayan Tiger	5/1	Bone, liver, feces/ muscle
***Panthera tigris tigris***	Bengal Tiger	1/1	Liver (FFPE*)/ muscle
***Panthera uncia***	Snow Leopard	11/14	Bone, skeletal muscle, feces/ heart, muscle, kidney, liver, bone
*Prionailurus bengalensis*	Leopard Cat	1/0	Lung/ NA^†^
*Puma concolor*	Puma	1/0	Liver/ NA^†^
*Panthera tigris*^Ψ^	Tiger	1	Mock tiger bone wine

Target species are in bold. Non-target species (non-bold) were used to test the primers for specificity. Results from experiments using these samples are presented in Tables 2 and 3, Tables S6–S8, Table S10, Figs. 3 and 4, and Figure S3.

*FFPE = Formalin-fixed, paraffin embedded tissue.

^†^NA = Not applicable.

Ψ = The sample was obtained from the Granby Zoo, Quebec, Canada, and was not a part of the WCS or the USFWS collections.

### DNA extraction from tissue, blood, feces, and tiger bone wine

Soft tissue samples were extracted with either the QIAamp® DNA Mini Kit or QIAcube HT kits (QIAGEN, Inc.) using the manufacturer’s protocol, or the PrepMan Ultra reagent. DNA extraction from soft tissue samples using PrepMan was performed by wiping tissue surfaces for 30 s with a sterile cotton swab. The swab was then added to 150 μl of PrepMan Ultra reagent, vortexed for 15 s, and incubated for 5 min at 95 °C. PCR inhibitors were removed by adding the PrepMan/sample lysate to a column using the OneStep™ PCR Inhibitor Removal Kit (Zymo Research) per manufacturer’s protocol. FFPE samples were extracted using a modified FFPE extraction protocol from the QIAamp DNA Mini and Blood Mini Handbook (see supplementary information for details). Blood sample extraction was performed using the QIAcube HT kit. Fecal samples were extracted with the QIAamp® DNA Stool Mini Kit (QIAGEN, Inc.) or the PrepMan rapid-extraction protocol. DNA was extracted from fecal samples using PrepMan by adding 100 mg of feces to 150 μl of PrepMan Ultra reagent and diluted 1:10 with nuclease free water after the PCR inhibition removal step. Mock tiger bone wine DNA extraction was performed using a modified FavorPrep Stool DNA Isolation Mini Kit protocol (FAVORGEN Biotech Corp.) (the bead beating step was not performed; see supplementary information for protocol details).

### DNA extraction from bone powder

A defleshed (warm water maceration) Malayan tiger (*P. tigris jacksoni*) skull was used in the development of our bone sample collection protocol. The target site for bone collection was first wiped with 10% bleach for one minute. A hand drill with a 5.5 mm cannulated drill bit (cut to a length of approximately 8 cm) was utilized to take a 100 mg core sample. The drill was operated at low speed (< 200 rpm) to mitigate potential DNA degradation from heat generated by the drill^36,37^. A metal stylet was used to push the bone core out of the drill bit and onto a bone morselizer (a tool commonly used in dentistry) where it was ground into small pieces. A sterile piece of paper folded into a funnel shape was used to transfer the ground bone from the morselizer into a 1.5 ml microcentrifuge tube for each sample. For samples where the drill was ineffective (*e.g*. teeth), a cordless Dremel® rotary tool equipped with a high-speed cutter bit was used at the lowest speed setting to shave off 100 mg of bone powder. All tools (drill, drill/Dremel® bits, metal stylet, and morselizer) were disinfected between processing each sample by soaking them in 50% bleach for 5 min, soaking in deionized water for one minute, rinsing with deionized water for 10 s, and drying them with paper towels. The inside of the drill bit was cleaned with 50% bleach using sterile cotton tipped applicators between each use.

For rapid DNA extraction from bone, the PrepMan Ultra™ Sample Preparation Reagent (with a PCR inhibition removal step) was used to extract DNA from bone powder. Briefly, 150 μl of the PrepMan Ultra Sample Preparation Reagent was added to 100 mg of bone powder, vortexed for 15 s, and incubated for 5 min at 95 °C. PCR inhibitors were removed by adding the PrepMan/bone powder lysate and adding it to a column using the OneStep™ PCR Inhibitor Removal Kit (Zymo Research) per manufacturers protocol. This entire DNA extraction process (from sample collection to final eluate) was completed in less than one hour.

The performance of the PrepMan protocol was compared to three standard commercial kits used for bone powder extractions: PureLink™ Genomic DNA Mini Kit (Invitrogen) with a modified protocol^38^, QIAGEN QIAamp® DNA Investigator Kit with manufacturer’s protocol (QIAGEN, Inc)^39^ couple with the demineralization protocol from Ewart et al. (2020), and QIAGEN QIAamp DNA Mini Kit with a user-developed bone extraction protocol^40^, replacing the demineralization step with the protocol from Ewart et al. (2020). Each DNA extraction protocol was used to extract nine replicate samples (three samples each from the parietal bone, jaw, and molar) resulting in a total of 36 extractions.

### Multiplex qPCR

Species-specific primers were designed for six big cat species: *Panthera tigris* (tiger), *Acinonyx jubatus* (cheetah), *Panthera pardus* (leopard), *Panthera leo* (lion), *Panthera uncia* (snow leopard), and *Panthera onca* (jaguar) (Supplementary Table S1). The primer sets targeted various mitochondrial genes (Table S1). Geneious Prime® version 2022.2.2 (https://www.geneious.com) was used to produce a whole-mitogenome nucleotide alignment of existing Felidae species available in GenBank (Supplementary Table S2). Primers were designed manually by maximizing nucleotide differences at potential primer binding sites between the target species and all other Felidae species in the alignment. Single-species primers were tested against DNA from target and off-target species (Table 1) to test each primer set for specificity. Primer sets were then combined incrementally with other species primer sets until a multiplex for all six target species was achieved.

Each 20 μl singleplex and multiplex PCR reaction included 10 μl of PowerUp™ SYBR™ Green Master Mix, 0.34 μl of primer mix (0.17 μM of each primer), either 1 μl (gBlock, tissue, FFPE) or 5 ul (bone, feces) of the associated DNA extract sample and either 8.66 μl or 4.66 μl molecular grade water, respectively. The qPCR cycling conditions included a 2-min hold at 50 °C for Uracil-DNA glycosylase (UDG; included in the PowerUp™ SYBR™ Green Master Mix) activation to prevent potential carryover contamination (contamination from previously amplified DNA), a 2-min hold at 95 °C , 40 cycles of [15 s at 95 °C and 60 s at 60 °C], and a high-resolution melt (HRM) step with 0.1 °C increments from 70 to 85 °C. Negative template controls (using molecular grade water in place of DNA) were used in all qPCR reactions.

PCR efficiency (a reflection of how well each set of primers amplify their DNA target) and sensitivity (the limit of detection for each set of primers)^41^ were assessed at each step in the validation process using synthetic oligonucleotides for each species (gBlocks™ gene fragments, Integrated DNA Technologies, Inc.; Supplementary Table S3). Each primer set was tested against a 1:10 serial dilution series of the corresponding gBlock DNA from each species, from 5 copies/μl to 5 × 10^6^ copies/μl. Each dilution series was qPCR amplified with three technical replicates and the resulting Cq values (number of qPCR cycles needed to detect the fluorescence signal) were used to plot a standard curve. The slope of the standard curve was then used to calculate the PCR efficiency with the formula: 10^(−1/slope)−1)^ * 100^42^. Primer sets were tested and screened to obtain optimal PCR efficiency between 90 and 100% for each primer set^43^. PCR sensitivity was measured as the lowest DNA copy number that was reliably amplified by the primers in two out of three technical replicates.

PCR specificity (how well each set of primers amplify the target DNA without cross amplification of non-target DNA)^44^ was assessed in multiplex by testing DNA from soft tissues, formalin-fixed paraffin-embedded (FFPE) tissue samples, or feces from each of the six target big cat species, off-target Felidae species, domestic animals, and human. Each species was tested using two technical replicates. Any samples that displayed off-target amplification in the melt curve analysis were then amplified separately in individual singleplex qPCRs with the six different primer sets to confirm amplification and identify which primers were leading to off-target amplification.

### DNA copy number quantification

DNA copy numbers were quantified with the formula y = m * ln(x) + b^45^, where y = sample Cq value, m = slope of the standard curve, x = DNA copy number, and b = y-intercept of the standard curve. The equation x = e^^((Sample Cq − y intercept)/(slope))^, was used to calculate DNA copy number. A Shapiro–Wilk’s test was used to test for normal distribution of Cq values and DNA copy numbers. To test for a significant difference in Cq values or DNA copy numbers between the PrepMan protocol and each of the other protocols, an independent sample t-test (for normally distributed data) or a Wilcoxon rank sum test (for non-normally distributed data) was used. A Bonferroni correction^46^ was implemented to control for the higher rate of Type I errors when performing multiple comparisons, and the *p*-value was set at 0.0167 (*p *= α/n, α = 0.05, n = 3).

### Intraspecific variation in melt peak temperatures

To assess intraspecific variation in melt peak temperatures, melt peaks from multiple individuals of a single species were compared. Testing was performed using samples from both WCS and USFWS. Archived soft tissue (ST), blood, FFPE tissue, bone, or fecal samples were used from: 26 Amur tigers (*P. tigris altaica*; 2 skull, 17 ST, 2 FFPE, 5 feces), 6 Malayan tigers (1 skull, 2 ST, 3 feces), 2 Bengal tigers (*P. tigris tigris*; 1 ST, 1 FFPE), 3 tigers (subspecies unknown; ST), 15 leopards (1 bone, 5 ST, 9 blood), 3 Amur leopards (*P. pardus orientalis*; 1 ST, 2 blood), 1 Sind leopard (*Panthera pardus sindica*; ST), 4 lions (3 ST, 1 blood), 4 Transvaal lions (*P. leo krugeri*; 1 ST, 3 feces), 9 cheetahs (ST), 7 Southern African Cheetahs (*Acinonyx jubatus jubatus*: 1 ST, 6 blood), 23 snow leopards (3 skulls, 13 ST, 7 feces), 12 jaguars (1 FFPE tissue, 11 ST), and one Goldman’s jaguar (*Panthera onca goldmani*; ST). (Supplementary Table S4 lists each sample, the institution it came from (WCS/USFWS) and how it was extracted).

The samples were amplified with the multiplex qPCR protocol described above using two to five technical replicates. The average, standard deviation, and range of the melt peak temperatures for each species was calculated using two to five technical replicates per sample.

### Pilot testing of multiplex qPCR protocol

Pilot testing of the multiplex qPCR protocol was performed at the USFWS laboratory using archived bone and soft tissue samples from a USFWS reference collection (Table 1). Bone powder from either a skull plate or vertebrae of each of the four big cat species (tiger, cheetah, snow leopard, and jaguar) was collected using a dental burr to drill 100 mg of bone powder from each sample. DNA was extracted using the PrepMan rapid-extraction protocol and amplified with the multiplex qPCR protocol (as described above) using six technical replicates. The PCR efficiency and sensitivity were also evaluated (as described above). Soft tissue samples from the reference collection include 89 archived tissue samples from: tiger = 19, jaguar = 12, lion = 13, snow leopard = 13, cheetah = 15, leopard = 17.

For reference sample melt peak temperature analysis, a qPCR replicate was considered positive if its melt peak temperature fell within ± 0.2 °C of the average positive control reference (gBlock) melt peak temperature. A conservative threshold of ± 0.2 °C was chosen to allow the maximum degree of variation in melt peak temperature for all species while minimizing temperature overlap between species that would lead to false positives. Any replicate that was positive for a big cat species (fell within ± 0.2 °C) but different from its reference melt peak temperature was called false positive. Any replicate that amplified but did not produce a melt peak temperature that fell within ± 0.2 °C of any big cat species was labelled as inconclusive. Replicates that did not amplify at all were labelled as no amplification. Sixty-four replicates that produced peaks indicative of more than one species, and were verified as contamination with additional testing, were removed from the analysis. Add additional five replicates were removed because they yielded a melt peak indicative of one species but sequenced to another species.

Multiplex qPCR pilot testing was also performed on mock tiger bone wine at McGill University, Montreal, Quebec, Canada. Two milliliters of mock tiger bone wine were collected after 36, 150, and 294 days of steeping and extracted as described above. DNA amplification was performed using the multiplex qPCR assay described above using three technical replicates. The efficiency and sensitivity of the tiger primers were assessed using the same tiger gBlock gene fragment as described above (Supplementary Table S2). R version 1.4.1106^47^ was used for all statistical tests and R package ggplot2 version 3.3.5^48^ was used to create all figures.

## Results

### Development of a multiplex qPCR to detect big cat species

**Figure 1:**
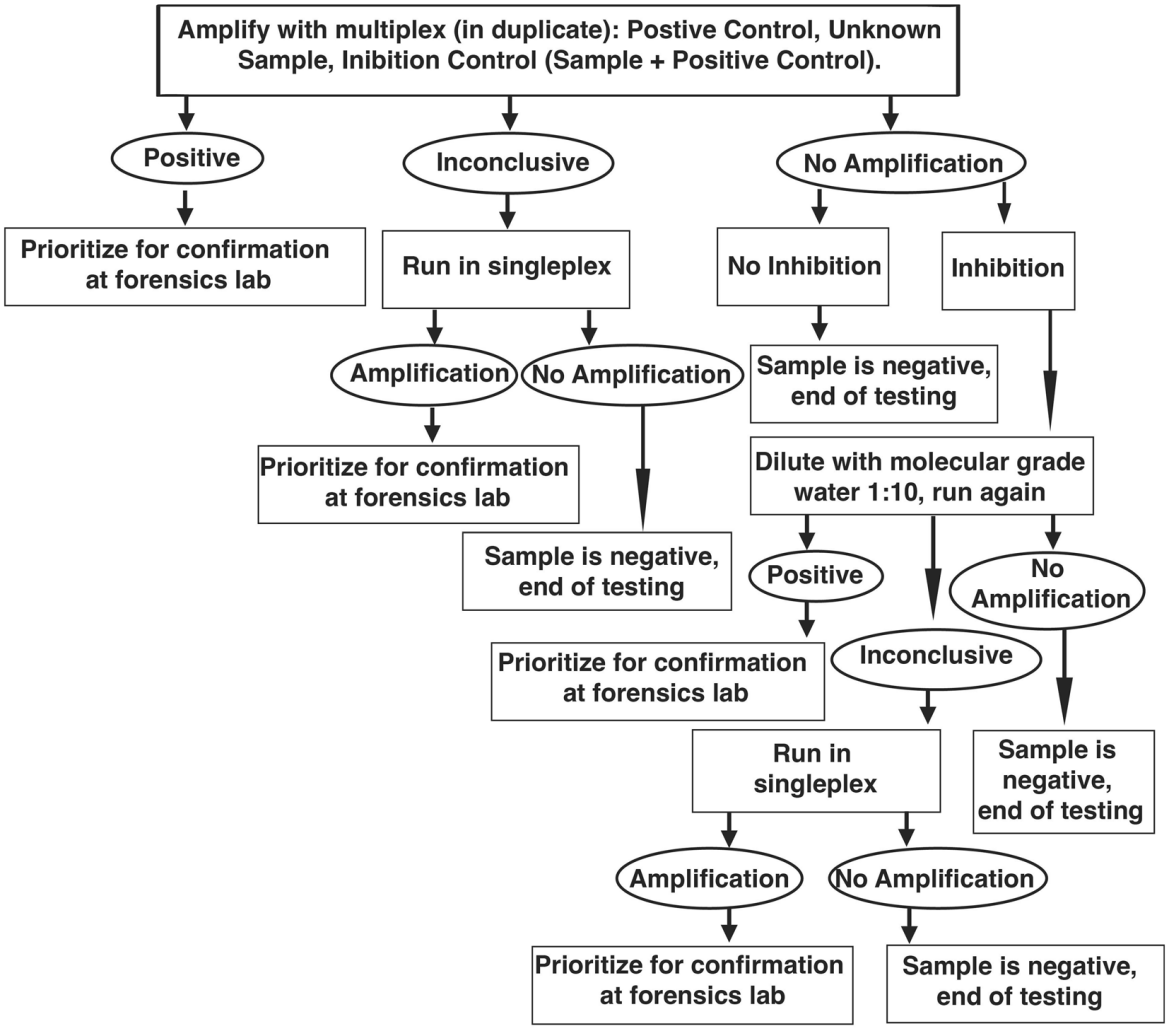
Recommended workflow for analysis and interpretation of results from unknown samples in the field.

**Figure 2:**
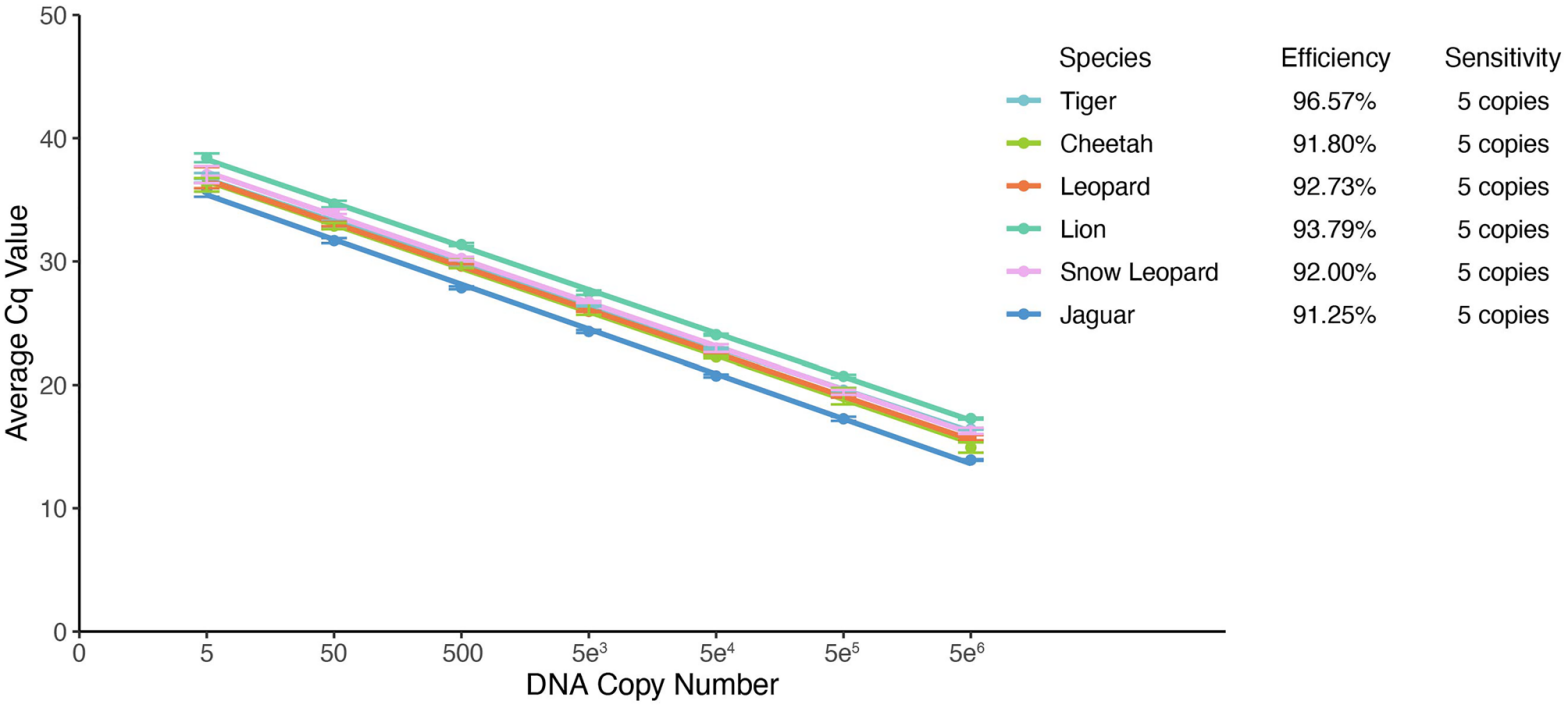
Primer set PCR efficiency (doubling of target DNA every round of amplification) and sensitivity (copy number limit of detection) for all six big cat species in the multiplex qPCR assay. Error bars indicate standard deviations. Note the X-axis is in logarithmic scale.

We developed a multiplex qPCR test using six primer sets targeting the following big cats: tiger, cheetah, leopard, lion, snow leopard, and jaguar. Figure 1 shows the recommended workflow for testing unknown samples. When tested against a positive control dilution series (gBlocks) in multiplex, all primer sets had PCR efficiencies that ranged from 91.3 to 96.6% and were sensitive down to 5 copies of DNA per PCR reaction (Fig. 2; see Supplementary Table S5). Similar results were also obtained when tested in the USFWS Forensic Laboratory (PCR efficiency ranged from 94.7 to 108.1% and were sensitive down to 5 copies per PCR reaction, see Supplementary Table S6).

All six primer sets were also examined for specificity against a panel of off-target species using synthetic or archived positive control material that had been sequenced to confirm species identity (Table 2). None of the six primers sets cross-amplified any of the other big cat species. However, DNA from one non-target species sample, a black-footed cat (*Felis nigripes*), amplified in the multiplex test. These results are discussed in more detail below.

**Table 2:** Testing target and off-target amplification by individual, species-specific primer sets. Cells marked with + signify positive amplification based on Cq values, and cells marked with –indicate no amplification.

	Lion primer set	Tiger primer set	Jaguar primer set	Leopard primer set	Snow Leopard primer set	Cheetah primer set
***Panthera leo***	** + **	–	–	–	–	–
***Panthera tigris altaica***	–	** + **	–	–	–	–
***Panthera tigris jacksoni***	–	** + **	–	–	–	–
***Panthera tigris tigris***	–	** + **	–	–	–	–
***Panthera onca***	–	–	** + **	–	–	–
***Panthera pardus***	–	–	–	** + **	–	–
***Panthera pardus orientalis***	–	–	–	** + **	–	–
***Panthera uncia***	–	–	–	–	** + **	–
***Acinonyx jubatus***	–	–	–	–	–	** + **
*Bos taurus*	–	–	–	–	–	–
*Caracal caracal*	–	–	–	–	–	–
*Canis familiaris*	–	–	–	–	–	–
*Equus caballus*	–	–	–	–	–	–
*Felis catus*	–	–	–	–	–	–
*Felis negripe*s	–	–	–	** + **	–	–
*Felis silvestris*	–	–	–	–	–	–
*Homo sapiens*	–	–	–	–	–	–
*Lynx canadensis*	–	–	–	–	–	–
*Leopardus pardalis*	–	–	–	–	–	–
*Lynx rufus	–	–	–	–	–	–
*Neofelis nebulosa*	–	–	–	–	–	–
*Ovis dalli*	–	–	–	–	–	–
*Otocolobus manul*	–	–	–	–	–	–
*Ovis vignei*	–	–	–	–	–	–
*Prionailurus bengalensis	–	–	–	–	–	–
*Puma concolor*	–	–	–	–	–	–

Samples in bold indicate big cat species targets in the multiplex assay.

* gBlocks were used in place of wildlife samples.

**Figure 3:**
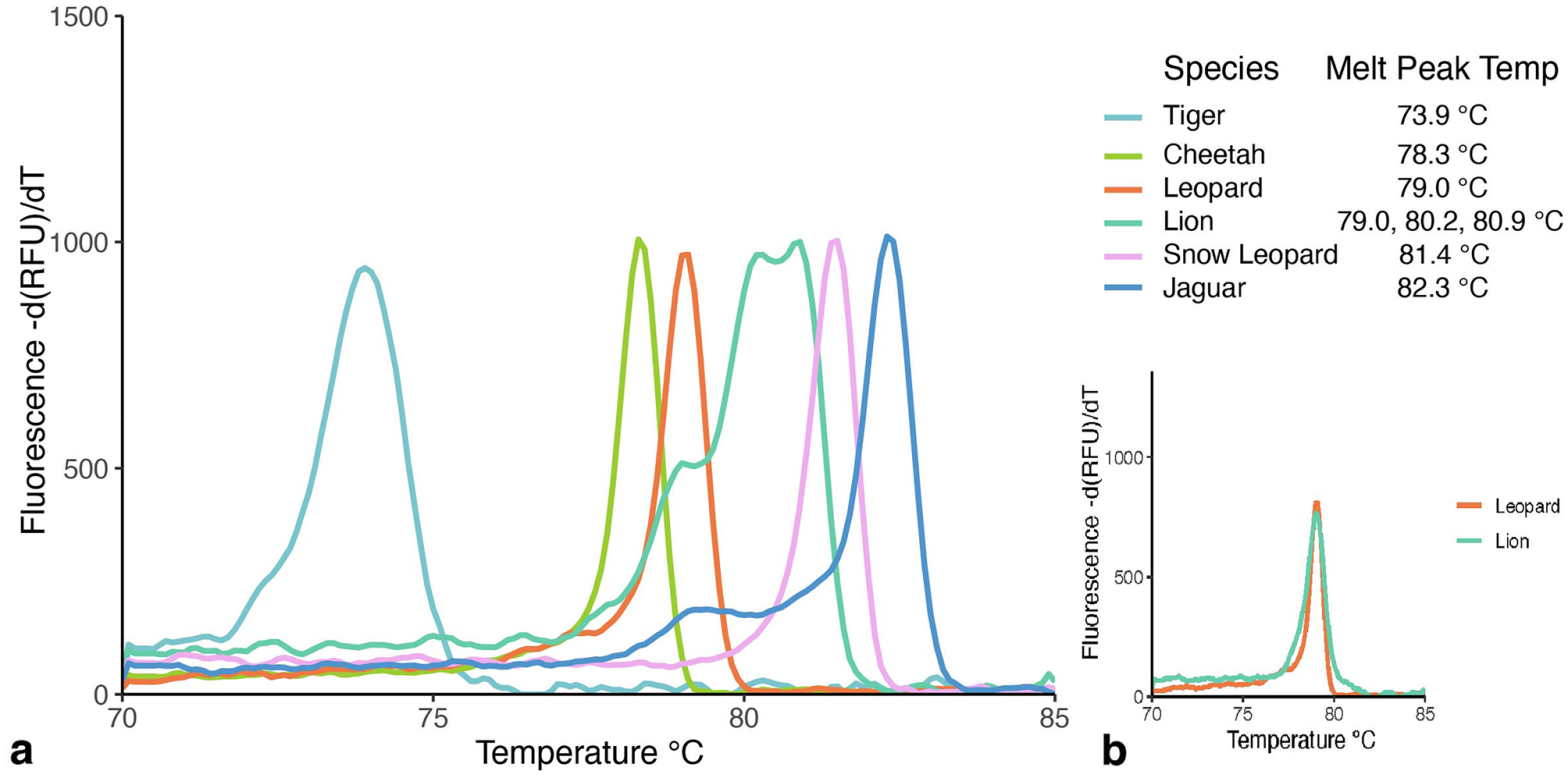
Expected melt temperature peak plot for target species in the multiplex qPCR test, based on the average melt peak temperatures of the gBlock positive controls. At high DNA copy number (> 50,000 copies) the lion amplicon produces multiple peaks due to cross amplification with a snow leopard primer, while only one melt peak (79.0 °C) is produced when DNA copy number is low (< 50,000; lower right inset). Figure inset shows the overlap of the lion and leopard melt peaks when lion is at low copy number. The plot displays the melt curve derivative with all peaks were standardized to the same height.

To test whether each of the six species could be differentiated from each other in the multiplex qPCR assay using melt peak temperature analysis, DNA from positive control gBlocks of each big cat species was amplified and the melt peak temperatures were averaged for each sample. All six species could be differentiated from each other by their species-specific melt peak temperatures when tested using gBlocks (Fig. 3). When testing the big cat multiplex qPCR using the gBlocks, we found that at high copy numbers the lion primers consistently produced a melt curve with multiple peaks that was easily distinguishable from the other species (> 50,000 copies, Cq = 17.22–24.22**)** (Fig. 2). Interestingly, this primer set produces a single peak that overlaps with the leopard melt peak at low copy numbers (< 50,000 copies, Cq = 27.46–34.68) (Fig. 3). Sanger sequencing followed by primer mapping identified where the multiple peaks originate from primer cross amplification. We found that the snow leopard forward primer cross-amplified lion DNA at high copy number leading to the multiple peaks (Supplementary Fig. S1). Cross-amplification does not occur with snow leopard DNA positive control in high or low concentration (Supplementary Figure S2a). Additionally, when adding equal number of copies of snow leopard and lion DNA, two separate and easily resolvable melt peaks of 79.0 °C and 81.4 °C are produced corresponding to snow leopard and lion respectively (Supplementary Figure S2b). These results indicated that the multiplex qPCR test can differentiate all six big cat species if DNA copy number is high, and can differentiate 4 of 6 (tiger, snow leopard, cheetah, jaguar) species at low DNA copy number (lion and leopard melt peaks overlap at low DNA copy number). We also determined that retesting the latter in singleplex with lion and leopard primers can resolve species identity.

Off-target amplification occurred with a black-footed cat DNA sample when amplified in the multiplex assay and produced two melt peaks that were within 0.5 °C of the average lion melt peaks. Upon additional testing with different primer combinations, it was determined that this off-target amplification was caused by the leopard forward and reverse primers (77.8 °C) and the snow leopard forward and lion reverse primer (80.7 °C). When samples were retested in singleplex with species-specific primer sets, the off-target melt peak of 80.7 °C disappeared but the 77.8 °C melt peak was not eliminated. However, this melt peak does not overlap with the leopard melt peak and therefore can be easily distinguished from leopard in singleplex.

### Testing a rapid DNA extraction method for DNA amplification from bone samples

To determine if PrepMan Ultra DNA extraction reagent could be used as an effective strategy to rapidly extract DNA from bone tissue to enable faster screening of bone products in the wildlife trade, we compared this extraction technique to standard bone extractions techniques that have been used by other studies^38,49,50^. Malayan tiger bone samples extracted with the PrepMan protocol produced Cq values between 33.2 and 39.0, corresponding to 0.84 to 44.88 copies of DNA/PCR reaction (see Supplementary Table S7 for data). Shapiro–Wilk tests of normality indicated normal distribution of Cq values (W = 0.978, *p *= 0.659) and non-normal distribution of DNA copy numbers (W = 0.598, *p *< 0.001). The PrepMan protocol had significantly higher Cq values than the PureLink (t = 3.814, *p *= 0.002) and the QIAamp user-developed (t = 5.078, *p *= 0.0001) protocols, and no significant difference was noted when compared to the QIAamp DNA Investigator Kit protocol (t = 2.075, *p *= 0.054) (Fig. S3a). Similarly, the QIAamp user-developed protocol (W = 77, *p *= 0.001), and the PureLink Kit protocol (W = 72.5, *p *= 0.005) had a significantly higher DNA copy number than the PrepMan protocol, whereas there was no significant difference between the PrepMan protocol and the QIAamp DNA Investigator Kit protocol (W = 65, *p *= 0.034) (Fig. S3b).

### Intraspecific melt peak temperature variation

**Figure 4:**
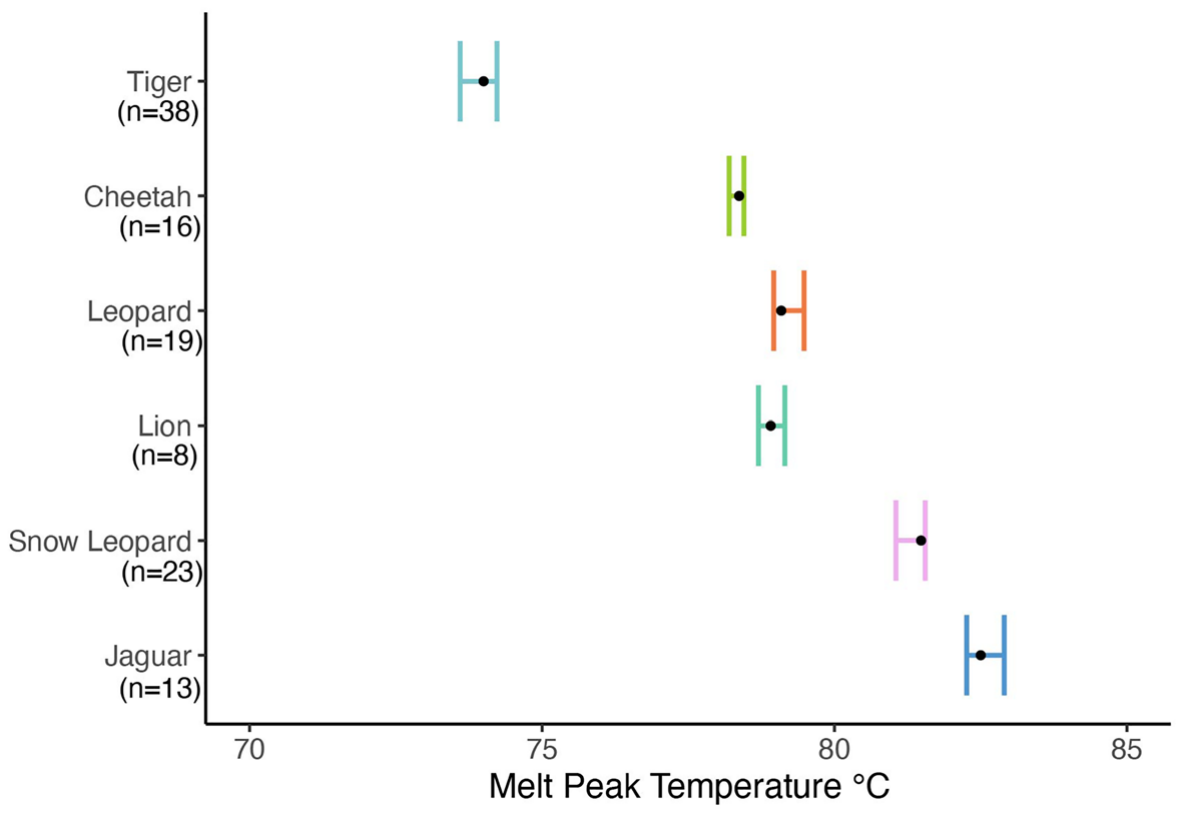
The variation in melt peak temperatures across the six big cat species. Average melt temperatures are represented as a black dot. Melt peak temperature ranges are displayed as colored bars. Subspecies of every species, except for snow leopard, were included in the study (Supplementary Table S4). n = number of individual animals/samples.

To determine how the multiplex qPCR test performs across individuals within a given species, we tested tissue samples from multiple individuals of each of the six big cat species (Table 1). The averages and ranges of melt peak temperatures for all six species are shown in Fig. 4 (see Supplementary Table S4 for more details). Melt peak temperatures varied across individuals within the same species from 0.25 to 0.64 °C. The largest standard deviation was 0.17 °C in jaguar. The multiplex qPCR test detected all four tiger subspecies (Amur, Bengal, Malayan, and Sumatran) and both the Amur and Sind leopards. Although the lion primer set produces multiple peaks in some samples, only one melt peak is reported in Fig. 4 (average of 78.91 °C) for lion in order to accommodate the variability in DNA copy number across samples.

### Pilot tests of the multiplex qPCR test

The large number of samples available in the USFWS reference collection, all with previously confirmed species identities (Supplementary Table S8), provided an opportunity to assess the percentage of true positive, false positive, and inconclusive results using the multiplex qPCR assay (Table 3). Of the 89 reference specimens, 92.30% were positive (correctly identified big cat species), 4.44% were inconclusive (could not be identified), 2.96% were false positive (DNA was amplified but the wrong species was identified), and 0.30% were not amplified (Table 3; see Supplementary Tables S8 and S9 for data). Positive identification for each species ranged from 84.09 (Jaguar) to 100% (Snow Leopard**)** (Table 3). Negative template controls were amplified in four out of fifty-four qPCR reactions. In all four instances the qPCRs were repeated and the negative controls were all negative.

**Table 3:** Big cat species identification of reference samples based on melt peak temperatures of each species (Tiger = 19, Cheetah = 15, Lion = 13, Leopard = 17, Snow Leopard = 13, Jaguar = 12).

Species	Tiger	Cheetah	Lion/Leopard	Snow Leopard	Jaguar	Total
Positive	69	54	99	53	37	312
	92.00%	98.18%	89.19%	100%	84.09%	92.31%
Inconclusive	5	1	4	0	5	15
	6.67%	1.82%	3.60%	0.00%	11.36%	4.44%
False Positive	1	0	7	0	2	10
	1.33%	0.00%	6.31%	0.00%	4.55%	2.96%
No Amplification	0	0	1	0	0	1
	0.00%	0.00%	0.90%	0.00%	0.00%	0.30%
Total	75	55	111	53	44	338

The numbers in the table indicate the number of replicates tested from each species and the percent of samples that were positive, inconclusive, false positive, or had no amplification. Lion and leopard results are combined because the melt peak temperatures from those species cannot be differentiated from each other.

DNA was also successfully amplified from four big cat bone samples (tiger, cheetah, snow leopard, jaguar). Cq values ranged from 23.70 to 34.13 for all four bone samples (Supplementary Table S6).

Testing was also performed to determine if the multiplex qPCR test could detect tiger DNA in mock tiger bone wine. Mock tiger bone wine samples were steeped for 36, 150, and 294 days and testing was performed in triplicate at McGill University. DNA was successfully amplified with the multiplex qPCR test (Cq values ranged from 33.31 to 35.33) in all mock tiger bone alcohol samples and steeping timepoints and produced melt peaks within the range for tiger (Supplementary Table S10). PCR efficiency of the tiger primers was 92.49% and the sensitivity was 5 copies (Supplementary Table S11 for data).

## Discussion

We developed a rapid, user-friendly point-of-care DNA test to detect the six most globally trafficked big cat species (lion, tiger, cheetah, snow leopard, jaguar, leopard). This multiplex qPCR test uses melt peak temperatures to identify and differentiate DNA from each target species. The six species-specific primer sets used in this assay do not cross-amplify DNA from the six target big cat species and did not amplify DNA from seventeen off-target species, including ten out of eleven non-target felid species. The only species for which the tested sample produced off-target amplification was from a black-footed cat. Amplified DNA produced two melt peaks that were within 0.5 °C of the average lion melt peak temperature in the multiplex assay. When tested in singleplex with each set of species-specific primers, black-footed cat DNA was amplified with the leopard primers and produced a single peak at 77.9 °C, which is more than 1.0 °C from the average leopard melt peak. Therefore, retesting any inconclusive samples (those that amplified but are not within ± 0.2 °C of the six big cat species) in singleplex would be a good strategy to confirm the initial multiplex qPCR test results.

We established that the PrepMan protocol takes less than an hour to complete and recovered enough DNA to consistently produce easily identifiable tiger-specific melt peaks from different bone locations. To our knowledge this is the first time that the PrepMan Ultra reagent has been used for the purpose of extracting DNA from bone. The PrepMan protocol was also successful in extraction of DNA from feces and tissue, consistent with other studies^51–53^. Despite the speed and ease-of-use of the PrepMan protocol, it recovered much less DNA when compared to the PureLink Genomic DNA Mini Kit with the Ewert et al. (2020) protocol and the QIAGEN QIAamp DNA Mini Kit with the user-developed bone extraction protocol. Thus, the optimal choice for DNA extraction method will ultimately depend on tradeoffs between the number of samples to be processed, sample quality, time constraints, and resource availability.

When assessing the intraspecific variation in melt peak temperature by amplifying DNA from different materials (tissue, blood, bone, feces) collected from multiple individuals, the maximum standard deviation in melt peak temperatures was 0.17 °C and the maximum range of melt peak temperatures was 0.64 °C. These results support the use of melt peak temperature as a means to differentiate big cat species. However, we noted that when lion DNA copy number is low (< 50,000 copies), its melt peak temperature overlapped with that of leopard, which prevented differentiation of the two species. Thus, any sample exhibiting a melt peak in the range for leopard should also be tested with individual lion and leopard species-specific primer sets for species confirmation. In addition, all big cat subspecies were detected by the multiplex qPCR, but the test does not differentiate between the subspecies. Additional experiments are needed to understand the consistency of the PCR assay for detecting animals across different geographical ranges and genetic ancestry, especially for wild populations that are most at risk for poaching and illegal trafficking.

Our results show that the multiplex qPCR test was highly sensitive and was able to detect the six big cat species down to five DNA copies/ PCR reaction. We also showed that PrepMan Ultra was successful in recovering DNA from four species of big cat bones and that the DNA was subsequently successfully amplified using the multiplex qPCR. It is interesting to note that some of the samples had been archived for more than 30 years. This indicates that our method is potentially applicable to samples that have been archived for a long time, enabling the possibility of retrospective forensic analysis to produce valuable historic data on big cat trade networks.

In addition, the multiplex qPCR test was able to amplify extracted tiger DNA from mock tiger bone wine samples steeped for 36, 150, and 295 days. Tiger bone wine is a major driver for illegal trafficking of tigers, and its use is deeply embedded within certain cultures where it is sought after for both its medicinal properties and as a status symbol^10^. Previous attempts to detect DNA from tiger bone wine have been inconclusive^16,54^. To our knowledge, this is the first time that DNA has been detected from any type of steeped alcohol including tiger bone wine, and it is promising that DNA was detected across all steeped timepoints, even those of relatively short duration.

Multiplex testing of reference tissue specimens with previously confirmed species identification resulted in an overall high positive rate with low false positive, inconclusive, and no amplification rates. However, 10 out of 338 samples had a false positive detection, such as jaguar identified as cheetah or snow leopard, or leopard identified as cheetah. Fifteen of 338 samples fell outside of the thresholds set for positive identification and were therefore inconclusive. False positives may occur due to environmental contamination of samples containing low DNA template. To minimize contamination, proper care should be taken to disinfect all tools with 50% bleach between samples. Positive samples as well as inconclusive samples can also be retested in singleplex to confirm results obtained in multiplex testing, and to rule out potential false positives. False negatives could be caused by DNA degradation and PCR inhibitors. In situations where more than one melt peak is observed, the results may indicate a mixed sample where two species are present, or a potential contamination event. In either case, retesting the sample in singleplex is recommended to confirm which species may be present in the sample, and if more than one species is present, then the user should consider extracting the sample and retesting in singleplex in replicate PCR reactions. Samples that do not amplify in the multiplex should be tested for PCR inhibition. One option is to dilute the extracted DNA with molecular grade water and retest the sample using the multiplex test with positive control spiked into the PCR reaction. In addition, we recommend retesting inconclusive samples in singleplex with each of the six big cat primer sets. See Fig. 1 for a suggested workflow.

This multiplex qPCR protocol has the potential to enable more efficient screening of wildlife trade products for six of the most trafficked big cat species in the world. It can be a useful tool at customs or border checkpoints to screen samples of bone, tissue, feces, and other processed products like traditional medicines as well as specimen types not directly tested in this study such as pelts/skins and claws. This method may be especially useful when materials cannot be identified by visual inspection (*e.g*. defleshed muscle or processed tissue, disarticulated long bones or vertebrae), incorporated into medicines or liquids (*e.g*. wine, medicinal tablets, powders) and when multiple big cat species are trafficked together or combined into a single product^55^. This rapid test can produce results that are available within hours, and is highly scalable and cost effective. The assay does not rely on more expensive chemistry such as Taqman probes, and the estimated cost per sample is on par with conventional PCR methods. Adaptation for use with portable thermocyclers^56^ for use in remote sites along supply chains (e.g. ports where shipping containers unload, mail facilities, and other transit hubs) could transform the enforcement community’s ability to disrupt wildlife crime networks. Use of our multiplex test can contribute to an improved understanding of the scale, scope, and use of big cat products that are trafficked globally and will inform education outreach and conservation efforts.

## Supplementary Information

Supplementary Information.

## Data Availability

All data generated or analyzed during this study are included in this published article (and its Supplementary Information files).
