# Supplementary material for: A new multiplex qPCR assay to detect and differentiate big cat species in the illegal wildlife trade
Source: Sci Rep. 2023 Jun 16;13:9796. doi: 10.1038/s41598-023-36776-z (PMC10275956; doi:10.1038/s41598-023-36776-z)
Supplement: Supplementary file 1 — Supplementary Information. [file 41598_2023_36776_MOESM1_ESM.docx]

**Supplementary Information**

Institutions that contributed samples to the USFWS Forensic Laboratory reference collection:

The Atlanta Zoo, The Brookfield Zoo and the Chicago Zoological Society, Chaffee Zoo, Charles Paddock Zoo, Cleveland Metroparks Zoo, Detroit Zoo, Erie Zoo, Gladys Porter Zoo, Idaho Department of Fish and Game, In-Sync Exotics Wildlife Rescue and Educational Center, John Ball Zoo, Kansas State University College of Veterinary Medicine, Lee Richardson Zoo, Lincoln Park Zoo, Louisville Zoo, Miami Metro 800, Milwaukee County Zoo, Minnesota Zoo, Oklahoma City Zoo, Oregon Zoo, Performing Animal Welfare Society, Philadelphia Zoo, Pittsburg Zoo, Sacramento Zoo, San Diego Wild Animal Park, San Diego Zoo, Santa Barbara Zoo, Tulsa Zoo, and the Woodland Park Zoo.

**DNA extraction from formalin-fixed, paraffin-embedded (FFPE) tissue**

1. Place tissue sections (not more than 25 mg) paraffin-embedded tissue in 1.5 ml

microcentrifuge tube.

1. Add 320 μl deparaffinization solution; vortex vigorously 10 s.
2. Incubate at 56 °C for 3 min, then allow to cool to RT.
3. Add 180 μl Buffer ATL; vortex to mix.
4. Centrifuge for 1 min at 10,000 rpm.
5. Add 20 μl proteinase K to the lower, clear phase; mix gently by pipetting up and down.
6. Incubate at 56 °C for 1 hour (or until sample is completely lysed).
7. Incubate at 90 °C for 1 hour.
8. Briefly centrifuge to remove drops from lid.
9. Transfer the lower, clearer phase into a new 2 ml tube.
10. Add 200 μl AL; pulse vortex 15 s.
11. Add 200 μl EtOH to sample; pulse vortex 15 s; centrifuge briefly.
12. Load QIAamp spin column in 2ml tube with entire volume of lysate.; close cap;

centrifuge (8,000 rpm)1 min; discard filtrate.

1. Place column in new collection.
2. Add 500 μl AW1; close cap; centrifuge (8,000 rpm) 1 min; discard filtrate.
3. Place column in new collection tube.
4. Add 500 μl AW2; close cap; centrifuge (8,000rpm) 3 min; discard filtrate.
5. Place column in new collection tube.
6. Centrifuge 3 min (14,000 rpm).
7. Transfer column to new collection tube; discard tube containing filtrate.
8. Add 50 μl dH2O; close tube, soak 1 min.
9. Centrifuge 1 min full speed (14,000 rpm).
10. Transfer to 1.5mL tube.

**DNA extraction from mock tiger bone wine**

1. Briefly vortex steeped alcohol sample.
2. Aliquot 2 mL of steeped alcohol into a 2 mL microcentrifuge tube.
3. Centrifuge for 30 minutes at 14,000 g.
4. Discard supernatant.
5. Air dry precipitate for one hour.
6. Extract DNA from dried precipitate using any standard fecal extraction protocol (e.g.

FavorPrep Stool DNA Isolation Mini Kit, QIAamp DNA Stool Mini Kit, GenElute Stool

DNA Isolation Kit, etc.), but skip the bead beating step.

1. Elute DNA using 50 μL of elution buffer and perform qPCR.

*If the user finds that after using these kits the sample still has PCR inhibition, this*

*optional step can be incorporated into this protocol.*

1. *PCR inhibitor removal (optional):* Prepare the PCR inhibitor removal spin columns by

placing the Zymo spin column into a Zymo collection tube.

1. Add 600 μL of the Zymo Prep Solution to each spin column.
2. Centrifuge spin columns at 8,000 x g for 3 minutes.
3. Discard the filtrate and again place the spin column on the collection tube and add 50 μL

of DNA to the spin column.

1. Centrifuge at 14,000 x g for 3 minutes.
2. Transfer the filtered solution to a 1.5 mL microcentrifuge tube.

**Table S1**. Big Cat Multiplex Primers: Gene and species target, length of amplified product, primer melt temperature, and primer sequences.

| Species | Primer Name | Gene  Target | Species  Detected | Length of  Amplicon (bp) | Primer  Tm (°C) | Primer Sequence (5’ –> 3’) |
| --- | --- | --- | --- | --- | --- | --- |
| Amur tiger  *(Panthera tigris)* | P. tig COI F | Cytochrome Oxidase C  Subunit 1 | *Panthera tigris altaica*, *Panthera tigris jacksoni, and Panthera tigris tigris* | 98 | 59.7 | TAGTTACTGCCCATGCCTTTGTGATAATCTTT |
|  | P. tig COI R |  |  |  | 56.9 | CTCCGATTATTAACGGAACTAGCCAG |
| Lion  *(Panthera leo)* | P. leo Cb F | Cytochrome B | *Panthera leo* | 152;  (425 & 700 at high copy number) | 55.1 | GAAATATTGGAATCATATTGTTGCTCACAG |
|  | P. leo Cb R |  |  |  | 64.0 | CCACTCTACTAGGTCGGCCCCGATG |
| Jaguar  *(Panthera onca)* | P. onca ATP6 F | ATP Synthase Membrane  Subunit 6 | *Panthera onca* | 463 | 57.4 | ACCCATTGTCATACTAATTATTATATTCCCCAGT |
|  | P. onca ATP6 R |  |  |  | 54.3 | GATTAAGTGCATTAATAAGTGGCCC |
| Leopard  *(Panthera pardus)* | P. pardus ND4 F | NADH Dehydrogenase  Subunit 4 | *Panthea pardus, Panthera pardus orientalis, and Felis negripes* | 364 | 56.8 | AATACTCATCATCTTGCAGCTTCTCT |
|  | P. pardus ND4 R |  |  |  | 61.7 | CGGTAGCCATAGGTGGAGTCCATATAGG |
| Cheetah  *(Acinonyx jubatus)* | A. jub COI F | Cytochrome Oxidase C  Subunit 1 | *Acinonyx jubatus* | 358 | 57.6 | GGCTAACTCTTCACTAGATATTGTTCTCCAT |
|  | A. jub COI R |  |  |  | 58.7 | AAAGGCTTCTCATACCATGAAAACTATAAG |
| Snow Leopard  *(Panthera uncia)* | P. uncia Cb F | Cytochrome B | *Panthera uncia* | 314 | 65.3 | TCCCACTCCATCCAACATCTCCGCATGATGA |
|  | P. uncia Cb R |  |  |  | 63.5 | TAGCCATGACTGCGAGCAATAGTACGGC |

Table S2. Primer testing: Whole mitogenomes used to test for potential primer binding sites in target and off-target species.

| Scientific Name | Common Name | GenBank Accession # |
| --- | --- | --- |
| *Acinonyx jabatus* | cheetah | KP202271 |
| *Caracal caracal* | caracal | KP202272 |
| *Catopuma badia* | bay cat | KP202256 |
| *Catopuma temminckii* | Asian golden cat | KX224509 |
| *Felis bieti* | Chinese mountain cat | KP202273 |
| *Felis catus* | domestic cat | NC_001700 |
| *Felis chaus* | jungle cat | KP202274 |
| *Felis margarita* | sand cat | KR132580 |
| *Felis nigripes* | black-footed cat | KP202277 |
| *Felis silvestris* | European wildcat | NC_028310 |
| *Homo sapiens* | human | NC_012920 |
| *Leopardus colocolo* | Pampas cat | KP202282 |
| *Leopardus geoffroyi* | Geoffroy’s cat | KP202292 |
| *Leopardus guigna* | kodkod | KP202293 |
| *Leopardus jacobita* | Andean mountain cat | KP202294 |
| *Leopardus pardalis* | ocelot | NC_028315 |
| *Leopardus tigrinus* | oncilla | KP202288 |
| *Leopardus wiedii* | margay | KP202289 |
| *Leptailurus serval* | serval | KP202286 |
| *Lynx canadensis* | Canada lynx | KP202281 |
| *Lynx lynx* | Eurasian lynx | KR132581 |
| *Lynx pardinus* | Iberian lynx | KX911411 |
| *Lynx rufus* | bobcat | GQ979707 |
| *Neofelis diardi* | Sunda clouded leopard | NC_072125 |
| *Neofelis nebulosa* | clouded leopard | KU133958 |
| *Otocolobus manul* | Pallas’s cat | KP202295 |
| *Panthera leo* | lion | KF907306 |
| *Panthera onca* | jaguar | KP202264 |
| *Panthera pardus* | leopard | NC_010641 |
| *Panthera pardus orientalis* | Amur leopard | KX655614 |
| *Panthera tigris* | tiger | JF357974 |
| *Panthera uncia* | snow leopard | KP202269 |
| *Pardofelis marmorata* | marbled cat | KT288227 |
| *Prionailurus bengalensis* | leopard cat | JN392459 |
| *Prionailurus planiceps* | flat-headed cat | KY682735 |
| *Prionailurus rubiginosus* | rusty-spotted cat | KP202266 |
| *Prionailurus viverrinus* | fishing cat | NC_028305 |
| *Profelis aurata* | African golden cat | KP202255 |
| *Puma concolor* | Cougar | MH814706 |
| *Puma yagouaroundi* | Jaguarundi | NC_028311 |

Table S3. Positive Control gBlocks, sequences, length and GenBank accession numbers for where the gBlock sequences were obtained. Melt peak temperatures for these controls should be established when running this assay for the first time in any laboratory. Melt peak temperatures can vary depending on the qPCR machine and PCR conditions.

| Species | Length (bp) | GenBank  Assession # | gBlock Sequence (5’ –> 3’) |
| --- | --- | --- | --- |
| Tiger  *Panthera tigris* | 136 | JF357974 | AAATTTATAATGTAGTAGTTACTGCCCATGCCTTTGTGATAATCTTTTTTATAGTAATGCCTATTAT  AATTGGAGGATTCGGAAACTGGCTAGTTCCGTTAATAATCGGAGCCCCCGATATGGCATTCCCTCG  AAT |
| Lion  *Panthera leo* | 467 | KF907306 | AACCACTCATTCATTGATCTTCCCACTCCACCCAATATCTCAGCATGATGAAACTTTGGCTCCTTAT  TAGGAGTATGTTTAATCCTACAAATTCTCACCGGCCTCTTTCTAGCCATACATTACACACCAGACAC  AATAACCGCTTTCTCATCAGTCACCCACATTTGCCGCGATGTAAACTATGGCTGAATTATCCGGTAC  CTACACGCCAACGGAGCCTCCATATTCTTTATCTGCCTATACATGCATGTAGGACGAGGAATATACT  ATGGCTCCTATACTTTCTCAGAAACATGAAATATTGGAATCATATTGTTGCTCACAGTTATAGCTAC  AGCCTTCATAGGATATGTCTTACCGTGGGGCCAAATATCCTTTTGAGGTGCAACTGTAATCACTAAT  CTCCTATCAGCAATCCCATACATCGGGGCCGACCTAGTAGAGTGGATCTGAGGAGGCTTCTCAGT |
| Jaguar  *Panthera onca* | 482 | KP202264 | GGACTACCCATTGTCATACTAATTATTATATTCCCCAGTATTCTATTCCCCTCGCCCAGTCGACTAAT  TAACAATCGTCTAATCTCACTCCAACAGTGATTAGTACAATTAACATCAAAACAAATACTAGCCATT  CACAACCACAAAGGGCAAACTTGGGCTCTAATACTCATATCTCTCATTCTATTCATTGGATCAACAA  ACCTGTTGGGCCTACTGCCCCACTCATTTACCCCAACTACCCAATTATCAATAAACTTAGGAATAGC  TATTCCCCTATGAGCCGGCACCGTAATTACTGGGTTTCGCCACAAAACTAAAGCATCCCTAGCCCAC  TTTCTACCACAAGGGACACCGATCCCCCTGATCCCCATGCTTGTAATTATTGAAACTATTAGCCTTTT  CATCCAACCCGTGGCTCTGGCCGTACGACTTACAGCCAACATTACTGCGGGCCACTTATTAATGCAC  TTAATCGGAGG |
| Leopard  *Panthera pardus* | 374 | NC_010641 | CATCACAATACTCATCATCTTGCAGCTTCTCTTAATTATAACATTCACTGCCACAGAACTGATCATAT  TCTATATTCTATTTGAAGCCACATTAATCCCCACTCTCATCATCATTACTCGATGGGGCAATCAAACA  GAACGACTAAACGCTGGCCTATATTTTCTATTCTATACCCTGATAGGCTCACTGCCCCTTCTAGTCGC  ACTACTATACATTCAAAACACAACAGGGACTTTAAATTTTCTAGTCATCCAATACTGAGCCAAACCA  ATCTCAGCCACTTGATCTAACATTTTTCTCTGACTAGCATGCATAATAGCATTTATAGTAAAAATACC  CCTATATGGACTCCACCTATGGCTACCGAAAGCAC |
| Cheetah  *Acinonyx jubatus* | 368 | KP202271 | GTACTGGCTAACTCTTCACTAGATATTGTTCTCCATGATACATATTACGTAGTAGCCCATTTCCACTA  TGTCTTATCAATAGGAGCAGTATTCGCTATCATAGGAGGCTTCGTCCATTGATTCCCCTTATTCTCAG  GATACACTCTTGATAATACTTGGGCAAAAATTCACTTCACGATTATATTTGTAGGAGTCAACATAAC  ATTCTTCCCTCAACACTTCCTAGGCCTATCTGGAATGCCACGACGTTATTCTGATTACCCAGATGCAT  ATACAACTTGAAACACAATTTCCTCAATAGGCTCTTTCATTTCATTAACAGCAGTCATACTTATAGTT  TTCATGGTATGAGAAGCCTTTGCATCCAA |
| Snow Leopard  *Panthera uncia* | 324 | KP202269 | GATCTTCCCACTCCATCCAACATCTCCGCATGATGAAACTTTGGCTCCCTGTTAGGAGTATGTTTAATC  CTACAAATTCTCACCGGCCTCTTTCTAGCCATACACTATACATCAGACACAATAACCGCTTTCTCGTCA  GTCACCCACATCTGCCGCGACGTAAATTATGGCTGAATTATCCGATACCTACACGCCAACGGAGCCTC  CATATTCTTTATCTGCCTATACATACACGTAGGACGAGGAATGTACTACGGCTCCTACACCTTCTCAGA  AACATGAAACATTGGAGCCGTACTATTGCTCGCAGTCATGGCTACAGCC |
| Leopard Cat  *Prionailurus*  *Bengalensis*  Cheetah Primers | 378 | JN392459 | GAATTGTACTAGCAAACTCCTCATTAGATATCGTTCTTCACGATACATATTATGTAGTAGCCCACTTCCAC  TATGTCTTGTCAATAGGAGCAGTATTCGCTATTATAGGAGGCTTTGTTCACTGATTTCCCCTATTCTCAGG  ATATACTCTCGACAATACTTGGGCAAAAATTCATTTCACAATTATGTTCGTGGGTGTCAACATAACATTCTT  CCCTCAACACTTTTTAGGCCTATCTGGAATGCCACGACGTTACTCTGACTACCCAGATGCATATACAACTT  GAAATACGATCTCCTCAATGGGCTCTTTCATCTCACTAACGGCAGTTATATTAATAGTCTTCATAGTGTGAG  AAGCTTTCGCGTCTAAACGAG |
| Bobcat  *Lynx rufus*  Cheetah Primers | 378 | KR132584 | GAATTGTATTAGCAAACTCCTCATTAGATATTGTTCTTCACGATACATATTACGTAGTAGCCCACTTCCACTAC  GTCCTGTCAATAGGAGCAGTATTCGCTATCATGGGAGGCTTTGTCCACTGGTTCCCCCTATTCTCAGGGTAT  ACTCTGGATGATACTTGGGCAAAAATCCACTTCACAATTATATTTGTGGGTGTCAACATGACGTTCTTTCCTCA  GCATTTCCTAGGCCTATCAGGAATGCCGCGACGTTATTCTGACTACCCAGATGCATACACAACTTGAAACACA  ATTTCCTCAATAGGCTCTTTTATCTCATTAACGGCAGTTATATTAATGGTTTTCATAGTGTGAGAAGCTTTTGCA  TCCAAGCGAG |

**Table S4**. The results for Figure 4 of testing the intraspecific variation of melt temperature in all six species. The average melt peak temperature of two to five technical replicates is shown for each sample.

| **Sample Name** | **Species** | **Institution** | **DNA Extraction**  **Method** | **Melt Peak Temperature (℃)** |
| --- | --- | --- | --- | --- |
| 20180070 | Southern African Cheetah | WCS | PrepMan | 78.30 |
| B11153 | Cheetah | USFWS | QIAcube HT Kit | 78.30 |
| D11370 | Cheetah | USFWS | QIAcube HT Kit | 78.40 |
| F31053 | Cheetah | USFWS | QIAcube HT Kit | 78.40 |
| H20831 | Cheetah | USFWS | QIAcube HT Kit | 78.30 |
| K30809 | Cheetah | USFWS | QIAcube HT Kit | 78.30 |
| K31253 | Southern African Cheetah | USFWS | QIAcube HT Kit | 78.30 |
| K31255 | Southern African Cheetah | USFWS | QIAcube HT Kit | 78.40 |
| K31257 | Southern African Cheetah | USFWS | QIAcube HT Kit | 78.30 |
| K31259 | Southern African Cheetah | USFWS | QIAcube HT Kit | 78.50 |
| K31261 | Southern African Cheetah | USFWS | QIAcube HT Kit | 78.40 |
| K31263 | Southern African Cheetah | USFWS | QIAcube HT Kit | 78.40 |
| K31301 | Cheetah | USFWS | QIAcube HT Kit | 78.40 |
| N10639 | Cheetah | USFWS | QIAcube HT Kit | 78.40 |
| N10811 | Cheetah | USFWS | QIAcube HT Kit | 78.40 |
| N11311 | Cheetah | USFWS | QIAcube HT Kit | 78.40 |
| 19970399 | Lion | WCS | PrepMan | 79.00 |
| 20060436 | Lion | WCS | PrepMan | 79.00 |
| 20080552 | Lion | WCS | PrepMan | 78.70 |
| 20120884 | Transvaal Lion | WCS | PrepMan | 78.75 |
| 13141 | Transvaal Lion | WCS | PrepMan | 78.90 |
| 13142 | Transvaal Lion | WCS | PrepMan | 78.90 |
| 13140 | Transvaal Lion | WCS | PrepMan | 78.90 |
| J40571 | Lion | USFWS | QIAcube HT Kit | 79.10 |
| 20000539 | Leopard | WCS | PrepMan | 79.00 |
| M20220010 | Leopard | WCS | PrepMan | 79.30 |
| G10164 | Leopard | USFWS | QIAcube HT Kit | 79.10 |
| G10166 | Leopard | USFWS | QIAcube HT Kit | 79.10 |
| G20406 | Leopard | USFWS | QIAcube HT Kit | 79.00 |
| D10263 | Leopard | USFWS | QIAcube HT Kit | 79.10 |
| P10534 | Leopard | USFWS | QIAcube HT Kit | 79.00 |
| P10537 | Leopard | USFWS | QIAcube HT Kit | 79.00 |
| P10538 | Leopard | USFWS | QIAcube HT Kit | 79.00 |
| P10542 | Leopard | USFWS | QIAcube HT Kit | 79.00 |
| P10543 | Leopard | USFWS | QIAcube HT Kit | 79.00 |
| P10544 | Leopard | USFWS | QIAcube HT Kit | 79.00 |
| P10545 | Leopard | USFWS | QIAcube HT Kit | 79.00 |
| P10546 | Leopard | USFWS | QIAcube HT Kit | 79.00 |
| P10547 | Leopard | USFWS | QIAcube HT Kit | 79.40 |
| P10539 | Amur Leopard | USFWS | QIAcube HT Kit | 79.00 |
| P10540 | Amur Leopard | USFWS | QIAcube HT Kit | 79.00 |
| P10665 | Amur Leopard | USFWS | QIAcube HT Kit | 79.10 |
| H21067 | Sind Leopard | USFWS | QIAcube HT Kit | 79.00 |
| 198507292 | Jaguar | WCS | Modified FFPE protocol | 82.50 |
| A40957 | Jaguar | USFWS | QIAcube HT Kit | 82.60 |
| C20220 | Jaguar | USFWS | QIAcube HT Kit | 82.30 |
| C20224 | Jaguar | USFWS | QIAcube HT Kit | 82.30 |
| D10269 | Jaguar | USFWS | QIAcube HT Kit | 82.30 |
| D40177 | Jaguar | USFWS | QIAcube HT Kit | 82.60 |
| G21257 | Jaguar | USFWS | QIAcube HT Kit | 82.90 |
| J10870 | Jaguar | USFWS | QIAcube HT Kit | 82.30 |
| L20830 | Goldman’s Jaguar | USFWS | QIAcube HT Kit | 82.30 |
| N10768 | Jaguar | USFWS | QIAcube HT Kit | 82.40 |
| N10769 | Jaguar | USFWS | QIAcube HT Kit | 82.40 |
| N30933 | Jaguar | USFWS | QIAcube HT Kit | 82.30 |
| N40349 | Jaguar | USFWS | QIAcube HT Kit | 82.60 |
| E41336 | Tiger | USFWS | QIAcube HT Kit | 74.10 |
| J20139 | Tiger | USFWS | QIAcube HT Kit | 74.00 |
| L31074 | Tiger | USFWS | QIAcube HT Kit | 74.00 |
| 59494 | Amur Tiger | WCS | PrepMan | 73.90 |
| 20140718 | Amur Tiger | WCS | PrepMan | 73.90 |
| 201403925 | Amur Tiger | WCS | modified FFPE protocol | 73.90 |
| 20150373 | Amur Tiger | WCS | PrepMan | 73.60 |
| 201604424 | Amur Tiger | WCS | modified FFPE protocol | 73.90 |
| 20120397 | Amur Tiger | WCS | PrepMan | 73.85 |
| 20130031 | Amur Tiger | WCS | PrepMan | 73.80 |
| 20150624 | Amur Tiger | WCS | PrepMan | 73.90 |
| 10076 | Amur Tiger | WCS | PrepMan | 74.00 |
| 10078 | Amur Tiger | WCS | PrepMan | 74.10 |
| 10077 | Amur Tiger | WCS | PrepMan | 74.00 |
| 15164 | Amur Tiger | WCS | PrepMan | 73.90 |
| 12091 | Amur Tiger | WCS | PrepMan | 74.05 |
| B11043 | Amur Tiger | USFWS | QIAcube HT Kit | 74.00 |
| B30631 | Amur Tiger | USFWS | QIAcube HT Kit | 73.90 |
| B30634 | Amur Tiger | USFWS | QIAcube HT Kit | 73.90 |
| C40349 | Amur Tiger | USFWS | QIAcube HT Kit | 74.00 |
| E20760 | Amur Tiger | USFWS | QIAcube HT Kit | 74.10 |
| G10718 | Amur Tiger | USFWS | QIAcube HT Kit | 74.00 |
| I30839 | Amur Tiger | USFWS | QIAcube HT Kit | 74.00 |
| I41024 | Amur Tiger | USFWS | QIAcube HT Kit | 74.00 |
| N10927 | Amur Tiger | USFWS | QIAcube HT Kit | 73.90 |
| H10342 | Amur Tiger | USFWS | QIAcube HT Kit | 74.00 |
| H10415 | Amur Tiger | USFWS | QIAcube HT Kit | 74.00 |
| K30802 | Amur Tiger | USFWS | QIAcube HT Kit | 74.00 |
| K40147 | Amur Tiger | USFWS | QIAcube HT Kit | 74.00 |
| 20140718 | Malayan Tiger | WCS | PrepMan | 73.90 |
| 20190880 | Malayan Tiger | WCS | PrepMan | 73.90 |
| 21017 | Malayan Tiger | WCS | PrepMan | 73.95 |
| 15154 | Malayan Tiger | WCS | PrepMan | 74.10 |
| 16001 | Malayan Tiger | WCS | PrepMan | 74.00 |
| N30772 | Malayan Tiger | USFWS | QIAcube HT Kit | 74.10 |
| 197901831 | Bengal Tiger | WCS | modified FFPE protocol | 73.90 |
| E41366 | Bengal Tiger | USFWS | QIAcube HT Kit | 74.10 |
| 20190379 | Snow Leopard | WCS | PrepMan | 81.50 |
| 2021113 | Snow Leopard | WCS | PrepMan | 81.05 |
| 17062 | Snow Leopard | WCS | PrepMan | 81.55 |
| 17159 | Snow Leopard | WCS | PrepMan | 81.50 |
| 19009 | Snow Leopard | WCS | PrepMan | 81.50 |
| 18153 | Snow Leopard | WCS | PrepMan | 81.45 |
| 19038 | Snow Leopard | WCS | PrepMan | 81.50 |
| 13047 | Snow Leopard | WCS | PrepMan | 81.50 |
| 14044 | Snow Leopard | WCS | PrepMan | 81.50 |
| 11136 | Snow Leopard | WCS | PrepMan | 81.25 |
| C21142 | Snow Leopard | USFWS | QIAcube HT Kit | 81.50 |
| D10318 | Snow Leopard | USFWS | QIAcube HT Kit | 81.40 |
| D30372 | Snow Leopard | USFWS | QIAcube HT Kit | 81.50 |
| D41147 | Snow Leopard | USFWS | QIAcube HT Kit | 81.50 |
| F10563 | Snow Leopard | USFWS | QIAcube HT Kit | 81.50 |
| F10565 | Snow Leopard | USFWS | QIAcube HT Kit | 81.50 |
| F10867 | Snow Leopard | USFWS | QIAcube HT Kit | 81.50 |
| F10869 | Snow Leopard | USFWS | QIAcube HT Kit | 81.50 |
| F10872 | Snow Leopard | USFWS | QIAcube HT Kit | 81.50 |
| F10874 | Snow Leopard | USFWS | QIAcube HT Kit | 81.50 |
| H21226 | Snow Leopard | USFWS | QIAcube HT Kit | 81.40 |
| I10579 | Snow Leopard | USFWS | QIAcube HT Kit | 81.40 |
| P30654 | Snow Leopard | USFWS | QIAcube HT Kit | 81.40 |

**Table S5**. The Cq values from triplicate qPCR reactions using a serial dilution series of gBlocks for each species that were used to calculate PCR efficiencies for each primer set in Figure 1**.**

| **Tiger qBock in Multiplex 6/22/2021** | |  |  |  |  |
| --- | --- | --- | --- | --- | --- |
| **Copies** | **Cq value 1** | **Cq value 2** | **Cq value 3** | **Average** | **St Dev** |
| 5 | 37.17 | 36.95 | 36.68 | 36.93 | 0.25 |
| 50 | 33.23 | 33.17 | 32.76 | 33.05 | 0.26 |
| 500 | 30.10 | 29.83 | 29.59 | 29.84 | 0.26 |
| 5000 | 26.52 | 26.37 | 26.40 | 26.43 | 0.08 |
| 50000 | 22.99 | 22.88 | 22.97 | 22.95 | 0.06 |
| 500000 | 19.77 | 19.39 | 19.56 | 19.57 | 0.19 |
| 5000000 | 16.41 | 16.47 | 16.38 | 16.42 | 0.05 |
|  |  |  |  |  |  |
| **Lion gBlock in Multiplex 6/23/21** | |  |  |  |  |
| **Copies** | **Cq value 1** | **Cq value 2** | **Cq value 3** | **Average** | **St Dev** |
| 5 | 38.41 | 37.91 |  | 38.16 | 0.35 |
| 50 | 34.66 | 34.68 | 34.21 | 34.52 | 0.27 |
| 500 | 31.37 | 31.32 | 31.56 | 31.42 | 0.13 |
| 5000 | 27.46 | 27.57 | 27.81 | 27.61 | 0.18 |
| 50000 | 24.06 | 24.17 | 24.22 | 24.15 | 0.08 |
| 500000 | 20.70 | 20.62 | 20.87 | 20.73 | 0.13 |
| 5000000 | 17.28 | 17.22 | 17.37 | 17.29 | 0.08 |
|  |  |  |  |  |  |
| **Leopard gBlock in Multiplex 7/2/21** | |  |  |  |  |
| **Copies** | **Cq value 1** | **Cq value 2** | **Cq value 3** | **Average** | **St Dev** |
| 5 | 37.15 | 35.83 | 37.42 | 36.80 | 0.85 |
| 50 | 33.16 | 33.11 | 32.84 | 33.04 | 0.17 |
| 500 | 29.67 | 29.46 | 29.75 | 29.63 | 0.15 |
| 5000 | 25.92 | 25.99 | 25.94 | 25.95 | 0.04 |
| 50000 | 22.31 | 22.39 | 22.53 | 22.41 | 0.11 |
| 500000 | 19.12 | 18.99 | 19.27 | 19.13 | 0.14 |
| 5000000 | 15.50 | 15.74 | 15.93 | 15.72 | 0.22 |
|  |  |  |  |  |  |
| **Jaguar gBlock in Multiplex 6/23/21** | |  |  |  |  |
| **Copies** | **Cq value 1** | **Cq value 2** | **Cq value 3** | **Average** | **St Dev** |
| 5 | 35.98 | 35.18 | 36.28 | 35.81 | 0.57 |
| 50 | 31.54 | 31.73 | 31.95 | 31.74 | 0.21 |
| 500 | 28.27 | 28.42 | 28.18 | 28.29 | 0.12 |
| 5000 | 24.76 | 24.60 | 24.53 | 24.63 | 0.12 |
| 50000 | 21.22 | 20.99 | 21.07 | 21.09 | 0.12 |
| 500000 | 17.95 | 17.64 | 17.67 | 17.75 | 0.17 |
| 5000000 | 14.33 | 14.46 | 14.39 | 14.39 | 0.07 |
|  |  |  |  |  |  |
| **Cheetah gBlock in Multiplex 6/23/21** | | |  |  |  |
| **Copies** | **Cq value 1** | **Cq value 2** | **Cq value 3** | **Average** | **St Dev** |
| 5 | 36.37 | 35.60 | 36.67 | 36.21 | 0.55 |
| 50 | 32.94 | 33.08 | 32.59 | 32.87 | 0.25 |
| 500 | 29.66 | 29.58 | 30.27 | 29.84 | 0.38 |
| 5000 | 25.90 | 25.89 | 26.84 | 26.21 | 0.55 |
| 50000 | 22.25 | 22.36 | 22.14 | 22.25 | 0.11 |
| 500000 | 18.71 | 18.70 | 19.86 | 19.09 | 0.67 |
| 5000000 | 15.40 | 14.79 | 14.60 | 14.93 | 0.42 |
|  |  |  |  |  |  |
| **Snow Leopard gBlock in Multiplex 7/8/21** | | |  |  |  |
| **Copies** | **Cq value 1** | **Cq value 2** | **Cq value 3** | **Average** | **St Dev** |
| 5 | 36.36 | 37.04 | 37.75 | 37.05 | 0.70 |
| 50 | 34.14 | 33.82 | 34.16 | 34.04 | 0.19 |
| 500 | 30.17 | 30.15 | 30.40 | 30.24 | 0.14 |
| 5000 | 26.62 | 26.76 | 26.75 | 26.71 | 0.08 |
| 50000 | 22.69 | 22.91 | 23.31 | 22.97 | 0.31 |
| 500000 | 19.32 | 19.27 | 19.64 | 19.41 | 0.20 |
| 5000000 | 15.99 | 16.46 | 16.39 | 16.28 | 0.25 |

**Table S6**. The Cq values from the USFWS lab used to calculate PCR efficiencies and sensitivity of the six big cat primer sets using gBlock positive controls, and to determine DNA copy number of the bone samples that were amplified with the multiplex.

| **Bone and gBlock Cq values** | | |  |  |  |  |  |  |
| --- | --- | --- | --- | --- | --- | --- | --- | --- |
| Species | Material | Copies | Cq1 | Cq2 | Cq3 | Cq4 | Cq5 | Cq6 |
| Tiger | bone | NA | 25.63 | 25.68 | 30.13 | 26.48 | 29.74 | 26.96 |
| Snow Leopard | bone | NA | 27.62 | 23.7 | 30.33 | 28.64 |  | 26.49 |
| Cheetah | bone | NA | 27.41 | 24.74 | 26.3 | 27.91 | 33.75 | 27.67 |
| Jaguar | bone | NA |  | 34.13 |  |  |  |  |
| Lion | gBlock | 5000000 | 12.53 | 13.41 | 13.63 |  |  |  |
| Lion | gBlock | 500000 | 15.8 | 16.07 | 16.35 |  |  |  |
| Lion | gBlock | 50000 | 19.02 | 19.35 | 19.61 |  |  |  |
| Lion | gBlock | 5000 | 21.8 | 22.35 | 23.05 |  |  |  |
| Lion | gBlock | 500 | 26.32 | 26.27 | 26.69 |  |  |  |
| Lion | gBlock | 50 | 29.23 | 29.04 | 29.59 |  |  |  |
| Lion | gBlock | 5 | 32.22 | 32.92 | 33.06 |  |  |  |
| Tiger | gBlock | 5000000 | 13.89 | 13.8 | 13.98 |  |  |  |
| Tiger | gBlock | 500000 | 17.24 | 17.11 | 17.35 |  |  |  |
| Tiger | gBlock | 50000 | 20.53 | 20.53 | 20.54 |  |  |  |
| Tiger | gBlock | 5000 | 24.13 | 24.05 | 23.97 |  |  |  |
| Tiger | gBlock | 500 | 27.31 | 27.26 | 27.43 |  |  |  |
| Tiger | gBlock | 50 | 30.89 | 30.8 | 30.67 |  |  |  |
| Tiger | gBlock | 5 | 33.66 | 33.62 | 34.14 |  |  |  |
| Cheetah | gBlock | 5000000 | 12.78 | 12.69 | 12.7 |  |  |  |
| Cheetah | gBlock | 500000 | 16.16 | 16.12 | 16.02 |  |  |  |
| Cheetah | gBlock | 50000 | 19.45 | 19.49 | 19.44 |  |  |  |
| Cheetah | gBlock | 5000 | 23.12 | 23 | 23.13 |  |  |  |
| Cheetah | gBlock | 500 | 26.32 | 26.41 | 26.19 |  |  |  |
| Cheetah | gBlock | 50 | 30.05 | 29.89 | 29.57 |  |  |  |
| Cheetah | gBlock | 5 | 33.58 | 33.78 | 32.87 |  |  |  |
| Leopard | gBlock | 5000000 | 16.05 | 16.05 | 16.7 |  |  |  |
| Leopard | gBlock | 500000 | 18.56 | 19.07 | 19.75 |  |  |  |
| Leopard | gBlock | 50000 | 21.95 | 22.2 | 23.99 |  |  |  |
| Leopard | gBlock | 5000 | 26 | 26.52 | 27.01 |  |  |  |
| Leopard | gBlock | 500 | 29.22 | 29.71 | 30.43 |  |  |  |
| Leopard | gBlock | 50 | 32.94 | 33.87 | 35.37 |  |  |  |
| Leopard | gBlock | 5 | 35.55 | 36.65 |  |  |  |  |
| Jaguar | gBlock | 5000000 | 19.75 | 18.18 | 18.77 |  |  |  |
| Jaguar | gBlock | 500000 | 21.66 | 20.67 | 21.48 |  |  |  |
| Jaguar | gBlock | 50000 | 25.02 | 24.09 | 25.08 |  |  |  |
| Jaguar | gBlock | 5000 | 27.85 | 27.65 | 28.58 |  |  |  |
| Jaguar | gBlock | 500 | 31.77 | 31.11 | 31.34 |  |  |  |
| Jaguar | gBlock | 50 | 35.48 | 34.56 | 33.78 |  |  |  |
| Jaguar | gBlock | 5 |  | 37.13 | 37.09 |  |  |  |
| Snow Leopard | gBlock | 5000000 | 10.83 | 12.07 | 11.43 |  |  |  |
| Snow Leopard | gBlock | 500000 | 13.65 | 14.47 | 14.91 |  |  |  |
| Snow Leopard | gBlock | 50000 | 17.08 | 17.5 | 18.05 |  |  |  |
| Snow Leopard | gBlock | 5000 | 20.83 | 21.22 | 21.5 |  |  |  |
| Snow Leopard | gBlock | 500 | 24.39 | 24.94 | 25.15 |  |  |  |
| Snow Leopard | gBlock | 50 | 27.74 | 28.15 | 27.96 |  |  |  |
| Snow Leopard | gBlock | 5 | 30.97 | 31.59 | 28.81 |  |  |  |


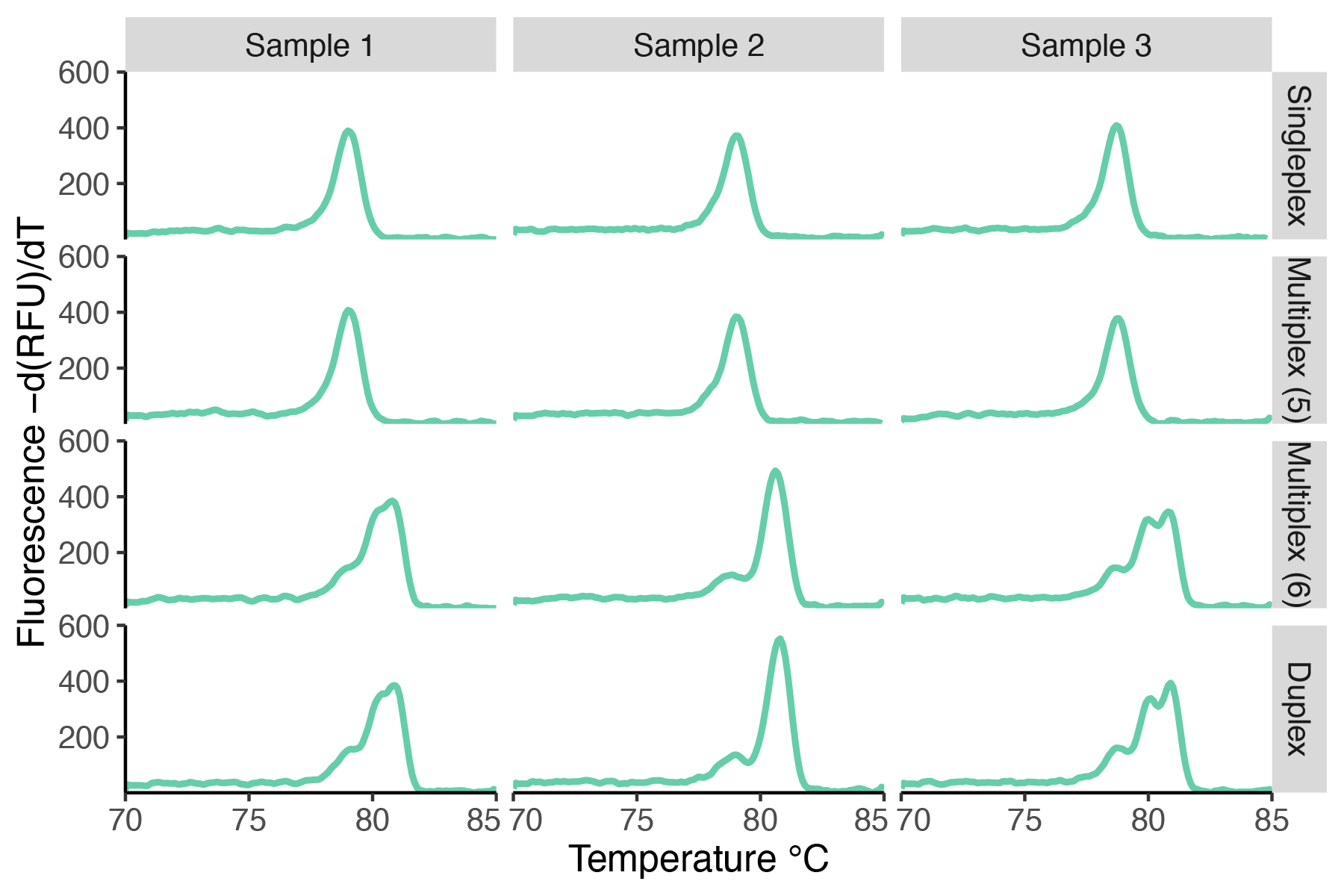


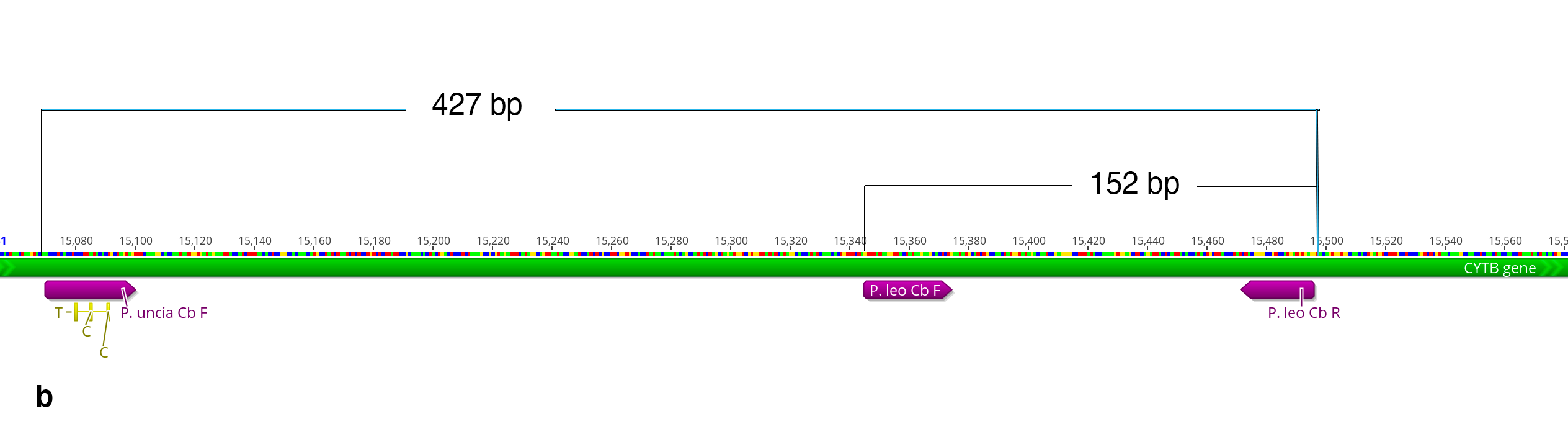


**Figure S1**. Multiple lion DNA peaks from Figure 2 occur only when both lion and snow leopard primers are present in the PCR reaction and when a high copy number of lion DNA (>50,000 copies) is present. a) Melt curve derivative plot of the three lion tissue samples (each sample >50,000 copies) in singleplex (lion primers only; top row labelled “Singleplex”), in multiplex excluding the snow leopard primers (second row labelled “Multiplex (5)”, with all six species (third row labelled “Multiplex (6)”, and with the snow leopard primers in duplex (bottom row labelled “Duplex”. Multiple melt peaks occur when lion DNA containing >50,000 DNA copies is amplified using primers in multiplex or in duplex with the lion and snow leopard primers (Supplementary Table S1). Only one peak is produced when amplified with the multiplex when snow leopard primers are excluded, or when tested in singleplex with only lion primers. b) Primer map created in Geneious Primer, version 2022.2.2, displaying the Cytochrome B gene region shows lion and snow leopard primers mapped to lion DNA sequence (GenBank No. KF907306). The yellow lines and associated nucleotides below snow leopard forward primer region represent the mismatches between the snow leopard forward primer and the lion DNA. The lion primer set amplifies a region of lion DNA that is 152 bp. In addition, the snow leopard forward primer and the leopard reverse primer amplify a lion DNA segment that is 427 bp.


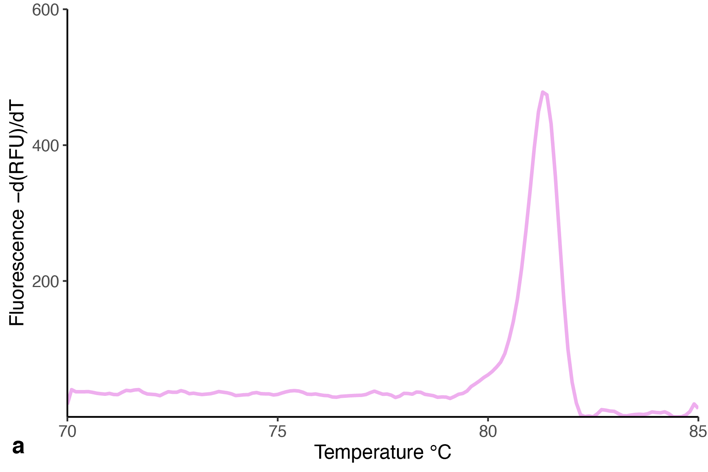

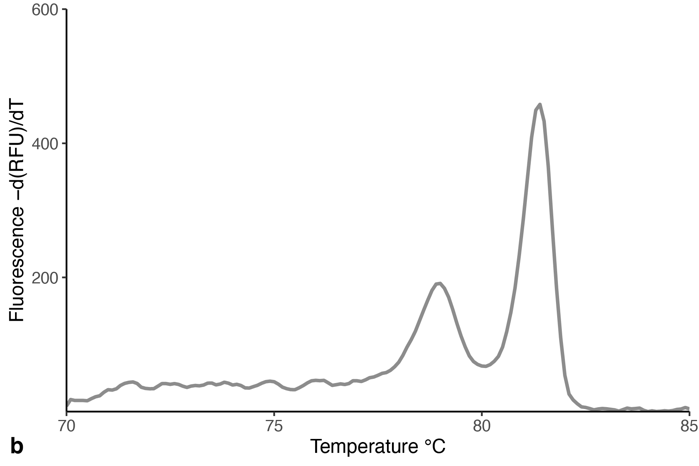


**Figure S2**. a) Only one peak at 81.4 °C is produced (no cross-amplification) when snow leopard gBlock DNA is amplified by the multiplex without lion DNA. b) When lion and snow leopard gBlock DNA (500,000 copies each) are added to the same tube, two separate peaks are produced at 79 °C and 81.4 °C, corresponding to the expected melt peaks for lion and snow leopard respectively.

**Table S7**. Data from comparison of the bone DNA extractions (Figure 3). Copy number results comparing the PrepMan rapid extraction protocol and commercial DNA extraction kits. These samples are also listed as part of Table 1.

| **DNA Extraction Method** | **Cq Value** | **y intercept** | **Slope** | **Copy Number** |
| --- | --- | --- | --- | --- |
| Tiger Jaw 1 Multiplex PrepMan | 38.60 | 38.75 | -1.459 | 1.11 |
| Tiger Jaw 2 Multiplex PrepMan | 36.60 | 38.75 | -1.459 | 4.36 |
| Tiger Jaw 3 Multiplex PrepMan | 36.80 | 38.75 | -1.459 | 3.81 |
| Tiger Molar 1 Multiplex PrepMan | 34.20 | 38.75 | -1.459 | 22.61 |
| Tiger Molar 2 Multiplex PrepMan | 36.90 | 38.75 | -1.459 | 3.55 |
| Tiger Molar 3 Multiplex PrepMan | 39.00 | 38.75 | -1.459 | 0.84 |
| Tiger Canine 1 Multiplex PrepMan | 35.00 | 38.75 | -1.459 | 13.07 |
| Tiger Canine 2 Multiplex PrepMan | 33.20 | 38.75 | -1.459 | 44.88 |
| Tiger Canine 3 Multiplex PrepMan | 36.60 | 38.75 | -1.459 | 4.36 |
| Tiger Jaw 1 Multiplex DNA Investigator | 34.70 | 38.75 | -1.459 | 16.05 |
| Tiger Jaw 2 Multiplex DNA Investigator | 33.90 | 38.75 | -1.459 | 27.78 |
| Tiger Jaw 3 Multiplex DNA Investigator | 34.50 | 38.75 | -1.459 | 18.41 |
| Molar 1 Multiplex DNA Investigator | 33.00 | 38.75 | -1.459 | 51.47 |
| Molar 2 Multiplex DNA Investigator | 38.30 | 38.75 | -1.459 | 1.36 |
| Molar 3 Multiplex DNA Investigator | 33.00 | 38.75 | -1.459 | 51.47 |
| Canine 1 Multiplex DNA Investigator | 32.50 | 38.75 | -1.459 | 72.51 |
| Canine 2 Multiplex DNA Investigator | 36.40 | 38.75 | -1.459 | 5.01 |
| Canine 3 Multiplex DNA Investigator | 34.10 | 38.75 | -1.459 | 24.22 |
| Tiger Jaw 1 Multiplex Purelink | 30.80 | 38.75 | -1.459 | 232.51 |
| Tiger Jaw 2 Multiplex Purelink | 34.10 | 38.75 | -1.459 | 24.22 |
| Tiger Jaw 3 Multiplex Purelink | 34.70 | 38.75 | -1.459 | 16.05 |
| Tiger Molar 1 Multiplex Purelink | 30.90 | 38.75 | -1.459 | 217.11 |
| Tiger Molar 2 Multiplex Purelink | 36.80 | 38.75 | -1.459 | 3.81 |
| Tiger Molar 3 Multiplex Purelink | 29.10 | 38.75 | -1.459 | 745.55 |
| Tiger Canine 1 Multiplex Purelink | 32.00 | 38.75 | -1.459 | 102.15 |
| Tiger Canine 2 Multiplex Purelink | 28.40 | 38.75 | -1.459 | 1204.60 |
| Tiger Canine 3 Multiplex Purelink | 32.20 | 38.75 | -1.459 | 89.07 |
| Tiger Jaw 1 Multiplex User Developed Bone Protocol | 30.75 | 38.75 | -1.459 | 241.44 |
| Tiger Jaw 2 Multiplex User Developed Bone Protocol | 29.92 | 38.75 | -1.459 | 425.00 |
| Tiger Jaw 3 Multiplex User Developed Bone Protocol | 29.42 | 38.75 | -1.459 | 598.72 |
| Tiger Canine 1 Multiplex User Developed Bone Protocol | 32.08 | 38.75 | -1.459 | 97.03 |
| Tiger Canine 2 Multiplex User Developed Bone Protocol | 32.27 | 38.75 | -1.459 | 85.18 |
| Tiger Canine 3 Multiplex User Developed Bone Protocol | 35.77 | 38.75 | -1.459 | 0.00 |
| Tiger Molar 1 Multiplex User Developed Bone Protocol | 33.26 | 38.75 | -1.459 | 43.07 |
| Tiger Molar 2 Multiplex User Developed Bone Protocol | 29.95 | 38.75 | -1.459 | 417.78 |
| Tiger Molar 3 Multiplex User Developed Bone Protocol | 31.44 | 38.75 | -1.459 | 149.95 |

**
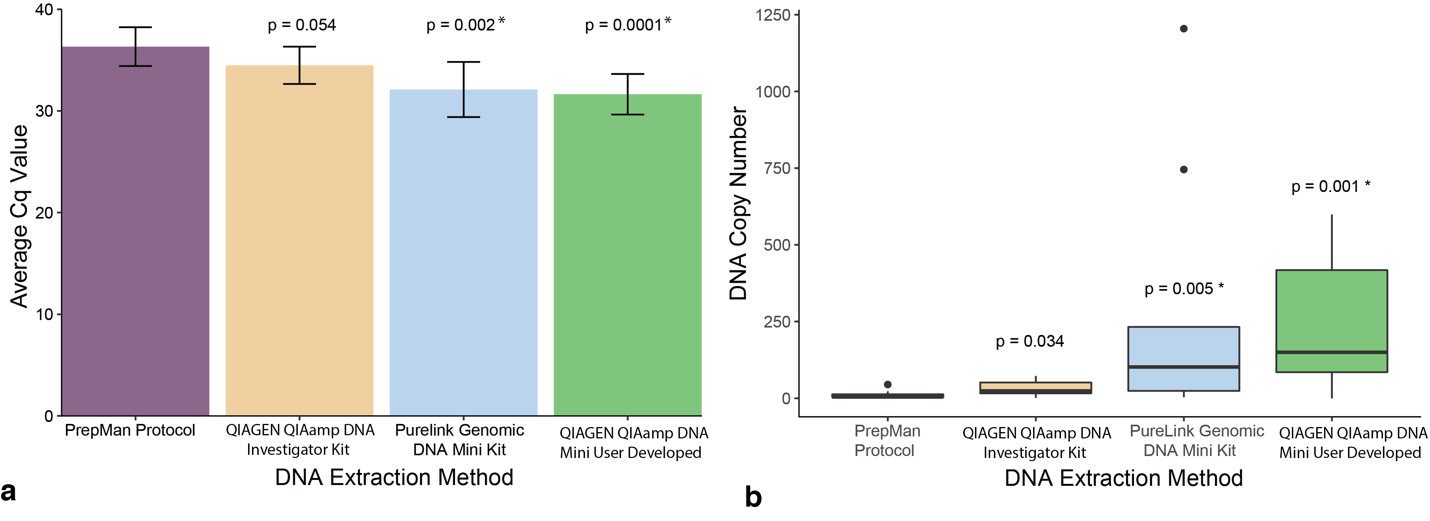
**

* represents a statistically significant difference compared to the PrepMan protocol (P<0.0167) after correction for multiple testing.

**Figure S3.** Comparison of a) average Cq values and b) DNA copy numbers/PCR reaction between the PrepMan protocol and other commercial DNA extraction kits for DNA detection of Malayan tiger DNA in a bone sample using the multiplex qPCR assay. Error bars represent standard deviations.

**Table S8**. Individual species identification from qPCR testing of reference samples in the USFWS laboratory that is summarized in Table 3. These samples are also listed as part of Table 1.

| Species | Sample Name | Melt Temperature | Called Species with +/- 0.2 ℃ |
| --- | --- | --- | --- |
| Cheetah | B11153 | 78.30 | Cheetah |
| Cheetah | B11153 | 78.30 | Cheetah |
| Cheetah | B11153 | 78.40 | Cheetah |
| Cheetah | B11153 | 78.40 | Cheetah |
| Cheetah | B11153 | 78.50 | Cheetah |
| Cheetah | D11370 | 78.40 | Cheetah |
| Cheetah | F31053 | 78.40 | Cheetah |
| Cheetah | F31053 | 78.40 | Cheetah |
| Cheetah | F31053 | 78.40 | Cheetah |
| Cheetah | H20831 | 78.30 | Cheetah |
| Cheetah | H20831 | 78.40 | Cheetah |
| Cheetah | H20831 | 78.40 | Cheetah |
| Cheetah | H20831 | 78.50 | Cheetah |
| Cheetah | K30809 | 78.30 | Cheetah |
| Cheetah | K30809 | 78.30 | Cheetah |
| Cheetah | K30809 | 78.40 | Cheetah |
| Cheetah | K30809 | 78.40 | Cheetah |
| Southern African Cheetah | K31253 | 78.30 | Cheetah |
| Southern African Cheetah | K31253 | 78.30 | Cheetah |
| Southern African Cheetah | K31253 | 78.40 | Cheetah |
| Southern African Cheetah | K31253 | 78.40 | Cheetah |
| Southern African Cheetah | K31255 | 78.30 | Cheetah |
| Southern African Cheetah | K31255 | 78.30 | Cheetah |
| Southern African Cheetah | K31255 | 78.30 | Cheetah |
| Southern African Cheetah | K31255 | 78.40 | Cheetah |
| Southern African Cheetah | K31257 | 78.30 | Cheetah |
| Southern African Cheetah | K31257 | 78.30 | Cheetah |
| Southern African Cheetah | K31257 | 78.30 | Cheetah |
| Southern African Cheetah | K31257 | 78.30 | Cheetah |
| Southern African Cheetah | K31257 | 78.40 | Cheetah |
| Southern African Cheetah | K31259 | 78.50 | Cheetah |
| Southern African Cheetah | K31259 | 78.40 | Cheetah |
| Southern African Cheetah | K31259 | 78.40 | Cheetah |
| Southern African Cheetah | K31261 | 78.40 | Cheetah |
| Southern African Cheetah | K31261 | 78.50 | Cheetah |
| Southern African Cheetah | K31261 | 78.40 | Cheetah |
| Southern African Cheetah | K31261 | 78.50 | Cheetah |
| Southern African Cheetah | K31263 | 78.40 | Cheetah |
| Southern African Cheetah | K31263 | 78.40 | Cheetah |
| Southern African Cheetah | K31263 | 78.40 | Cheetah |
| Southern African Cheetah | K31263 | 78.40 | Cheetah |
| Cheetah | K31301 | 78.40 | Cheetah |
| Cheetah | K31301 | 78.40 | Cheetah |
| Cheetah | K31301 | 78.40 | Cheetah |
| Cheetah | K31301 | 78.40 | Cheetah |
| Cheetah | N10639 | 78.40 | Cheetah |
| Cheetah | N10639 | 78.40 | Cheetah |
| Cheetah | N10639 | 78.50 | Cheetah |
| Cheetah | N10811 | 78.40 | Cheetah |
| Cheetah | N10811 | 78.40 | Cheetah |
| Cheetah | N10811 | 78.40 | Cheetah |
| Cheetah | N11311 | 78.40 | Cheetah |
| Cheetah | N11311 | 78.40 | Cheetah |
| Cheetah | N11311 | 78.40 | Cheetah |
| Cheetah | D11370 | 78.00 | Inconclusive |
| Jaguar | A40957 | 82.60 | Jaguar |
| Jaguar | C20220 | 82.30 | Jaguar |
| Jaguar | C20220 | 82.40 | Jaguar |
| Jaguar | C20220 | 82.40 | Jaguar |
| Jaguar | C20220 | 82.50 | Jaguar |
| Jaguar | C20224 | 82.30 | Jaguar |
| Jaguar | C20224 | 82.40 | Jaguar |
| Jaguar | C20224 | 82.40 | Jaguar |
| Jaguar | C20224 | 82.50 | Jaguar |
| Jaguar | C20224 | 82.50 | Jaguar |
| Jaguar | D10269 | 82.30 | Jaguar |
| Jaguar | D10269 | 82.40 | Jaguar |
| Jaguar | D10269 | 82.40 | Jaguar |
| Jaguar | D10269 | 82.40 | Jaguar |
| Jaguar | D10269 | 82.50 | Jaguar |
| Jaguar | D40177 | 82.60 | Jaguar |
| Jaguar | D40177 | 82.60 | Jaguar |
| Jaguar | J10870 | 82.30 | Jaguar |
| Jaguar | J10870 | 82.40 | Jaguar |
| Jaguar | J10870 | 82.40 | Jaguar |
| Goldman’s Jaguar | L20830 | 82.30 | Jaguar |
| Goldman’s Jaguar | L20830 | 82.40 | Jaguar |
| Goldman’s Jaguar | L20830 | 82.40 | Jaguar |
| Goldman’s Jaguar | L20830 | 82.40 | Jaguar |
| Jaguar | N10768 | 82.40 | Jaguar |
| Jaguar | N10768 | 82.50 | Jaguar |
| Jaguar | N10768 | 82.50 | Jaguar |
| Jaguar | N10769 | 82.40 | Jaguar |
| Jaguar | N10769 | 82.50 | Jaguar |
| Jaguar | N10769 | 82.50 | Jaguar |
| Jaguar | N30933 | 82.30 | Jaguar |
| Jaguar | N30933 | 82.40 | Jaguar |
| Jaguar | N30933 | 82.40 | Jaguar |
| Jaguar | N30933 | 82.40 | Jaguar |
| Jaguar | N40349 | 82.60 | Jaguar |
| Jaguar | N40349 | 82.60 | Jaguar |
| Jaguar | N40349 | 82.60 | Jaguar |
| Jaguar | A40957 | 82.70 | Inconclusive |
| Jaguar | D40177 | 82.70 | Inconclusive |
| Jaguar | D40177 | 82.70 | Inconclusive |
| Jaguar | G21257 | 82.90 | Inconclusive |
| Jaguar | G21257 | 82.90 | Inconclusive |
| Jaguar | N40349 | 78.30 | Cheetah |
| Jaguar | C20220 | 81.70 | Snow Leopard |
| Lion | C40521 | 80.90 | Lion |
| Lion | C40521 | 80.90 | Lion |
| Lion | C40521 | 81.00 | Lion |
| Lion | C40521 | 81.00 | Lion |
| Lion | C40521 | 81.00 | Lion |
| Lion | E40869 | 80.90 | Lion |
| Lion | E40869 | 81.00 | Lion |
| Lion | E40869 | 81.00 | Lion |
| Lion | E40869 | 81.00 | Lion |
| Lion | H30872 | 81.00 | Lion |
| Lion | H30872 | 81.00 | Lion |
| Lion | H30872 | 81.10 | Lion |
| Lion | H30872 | 81.10 | Lion |
| Transvaal Lion | J40420 | 81.00 | Lion |
| Transvaal Lion | J40420 | 80.80 | Lion |
| Transvaal Lion | J40420 | 80.90 | Lion |
| Lion | J40553 | 80.90 | Lion |
| Lion | J40553 | 81.10 | Lion |
| Lion | J40566 | 80.90 | Lion |
| Lion | J40566 | 81.00 | Lion |
| Lion | J40566 | 81.00 | Lion |
| Lion | J40566 | 81.00 | Lion |
| Lion | J40571 | 79.10 | L/L |
| Lion | J40571 | 79.10 | L/L |
| Lion | J40571 | 79.10 | L/L |
| Lion | J40571 | 79.30 | L/L |
| Lion | J40571 | 80.80 | Lion |
| Lion | K30232 | 80.90 | Lion |
| Lion | K30232 | 81.00 | Lion |
| Lion | K30232 | 81.00 | Lion |
| Lion | K30232 | 81.00 | Lion |
| Transvaal Lion | M30574 | 80.90 | Lion |
| Transvaal Lion | M30574 | 80.90 | Lion |
| Transvaal Lion | M30574 | 81.00 | Lion |
| Transvaal Lion | M30574 | 81.00 | Lion |
| Lion | M30808 | 81.00 | Lion |
| Lion | M30808 | 81.00 | Lion |
| Lion | M30808 | 81.00 | Lion |
| Lion | N11359 | 80.90 | Lion |
| Lion | N31026 | 80.80 | Lion |
| Lion | N31026 | 80.90 | Lion |
| Lion | N31026 | 80.90 | Lion |
| Lion | N31078 | 80.90 | Lion |
| Leopard | D10263 | 79.10 | L/L |
| Leopard | D10263 | 78.80 | L/L |
| leopard | D10263 | 78.90 | L/L |
| Leopard | D10263 | 78.90 | L/L |
| Leopard | D10263 | 79.00 | L/L |
| Leopard | D10263 | 79.00 | L/L |
| Leopard | D10263 | 79.00 | L/L |
| Leopard | G10164 | 79.10 | L/L |
| Leopard | G10164 | 79.10 | L/L |
| Leopard | G10164 | 79.10 | L/L |
| Leopard | G10166 | 79.10 | L/L |
| Leopard | G20406 | 79.00 | L/L |
| Leopard | G20406 | 79.10 | L/L |
| Leopard | G20406 | 79.10 | L/L |
| Leopard | G20406 | 79.20 | L/L |
| Amur Leopard | H10665 | 79.10 | L/L |
| Amur Leopard | H10665 | 79.10 | L/L |
| Amur Leopard | H10665 | 79.10 | L/L |
| Sindh Leopard | H21067 | 79.00 | L/L |
| Sindh Leopard | H21067 | 79.10 | L/L |
| Sindh Leopard | H21067 | 79.20 | L/L |
| Sindh Leopard | H21067 | 79.20 | L/L |
| Leopard | P10534 | 79.00 | L/L |
| Leopard | P10534 | 79.00 | L/L |
| Leopard | P10534 | 79.10 | L/L |
| Leopard | P10537 | 79.00 | L/L |
| Leopard | P10537 | 79.10 | L/L |
| Leopard | P10537 | 79.10 | L/L |
| Leopard | P10538 | 79.00 | L/L |
| Leopard | P10538 | 79.10 | L/L |
| Leopard | P10538 | 79.10 | L/L |
| Leopard | P10539 | 79.00 | L/L |
| Leopard | P10539 | 79.10 | L/L |
| Leopard | P10539 | 79.10 | L/L |
| Leopard | P10540 | 79.00 | L/L |
| Leopard | P10540 | 79.10 | L/L |
| Leopard | P10540 | 79.10 | L/L |
| Leopard | P10542 | 79.00 | L/L |
| Leopard | P10542 | 79.10 | L/L |
| Leopard | P10542 | 79.10 | L/L |
| Leopard | P10542 | 80.90 | L/L |
| Leopard | P10543 | 79.00 | L/L |
| Leopard | P10543 | 79.10 | L/L |
| Leopard | P10543 | 79.10 | L/L |
| Leopard | P10543 | 81.00 | L/L |
| Leopard | P10544 | 79.00 | L/L |
| Leopard | P10544 | 79.00 | L/L |
| Leopard | P10544 | 79.10 | L/L |
| Leopard | P10545 | 79.00 | L/L |
| Leopard | P10545 | 79.00 | L/L |
| Leopard | P10545 | 79.00 | L/L |
| Leopard | P10545 | 79.00 | L/L |
| Leopard | P10546 | 79.00 | L/L |
| Leopard | P10546 | 79.00 | L/L |
| Leopard | P10546 | 79.10 | L/L |
| Leopard | P10546 | 79.10 | L/L |
| Leopard | P10547 | 79.40 | Inconclusive |
| Leopard | P10547 | 79.40 | Inconclusive |
| Leopard | P10547 | 79.50 | Inconclusive |
| Leopard | P10547 | 79.60 | Inconclusive |
| Leopard | P10534 | 74.00 | Tiger |
| Leopard | P10539 | 74.00 | Tiger |
| Leopard | P10540 | 82.40 | Jaguar |
| Leopard | P10544 | 82.50 | Jaguar |
| Lion | N31078 | 82.30 | Jaguar |
| Lion | N31026 | 82.40 | Jaguar |
| leopard | P10537 | 78.30 | Cheetah |
| Lion | J40553 | None | No Amplification |
| Snow Leopard | C21142 | 81.50 | Snow Leopard |
| Snow Leopard | C21142 | 81.50 | Snow Leopard |
| Snow Leopard | C21142 | 81.60 | Snow Leopard |
| Snow Leopard | C21142 | 81.60 | Snow Leopard |
| Snow Leopard | C21142 | 81.40 | Snow Leopard |
| Snow Leopard | D10318 | 81.40 | Snow Leopard |
| Snow Leopard | D10318 | 81.50 | Snow Leopard |
| Snow Leopard | D10318 | 81.50 | Snow Leopard |
| Snow Leopard | D10318 | 81.60 | Snow Leopard |
| Snow Leopard | D10318 | 81.40 | Snow Leopard |
| Snow Leopard | D30372 | 81.50 | Snow Leopard |
| Snow Leopard | D30372 | 81.50 | Snow Leopard |
| Snow Leopard | D30372 | 81.60 | Snow Leopard |
| Snow Leopard | D30372 | 81.60 | Snow Leopard |
| Snow Leopard | D41147 | 81.50 | Snow Leopard |
| Snow Leopard | D41147 | 81.50 | Snow Leopard |
| Snow Leopard | D41147 | 81.60 | Snow Leopard |
| Snow Leopard | D41147 | 81.60 | Snow Leopard |
| Snow Leopard | F10563 | 81.50 | Snow Leopard |
| Snow Leopard | F10563 | 81.50 | Snow Leopard |
| Snow Leopard | F10563 | 81.50 | Snow Leopard |
| Snow Leopard | F10563 | 81.60 | Snow Leopard |
| Snow Leopard | F10565 | 81.50 | Snow Leopard |
| Snow Leopard | F10565 | 81.50 | Snow Leopard |
| Snow Leopard | F10565 | 81.50 | Snow Leopard |
| Snow Leopard | F10565 | 81.60 | Snow Leopard |
| Snow Leopard | F10867 | 81.50 | Snow Leopard |
| Snow Leopard | F10867 | 81.50 | Snow Leopard |
| Snow Leopard | F10867 | 81.60 | Snow Leopard |
| Snow Leopard | F10867 | 81.60 | Snow Leopard |
| Snow Leopard | F10869 | 81.50 | Snow Leopard |
| Snow Leopard | F10869 | 81.50 | Snow Leopard |
| Snow Leopard | F10869 | 81.50 | Snow Leopard |
| Snow Leopard | F10869 | 81.60 | Snow Leopard |
| Snow Leopard | F10872 | 81.50 | Snow Leopard |
| Snow Leopard | F10872 | 81.50 | Snow Leopard |
| Snow Leopard | F10872 | 81.50 | Snow Leopard |
| Snow Leopard | F10872 | 81.60 | Snow Leopard |
| Snow Leopard | F10874 | 81.50 | Snow Leopard |
| Snow Leopard | F10874 | 81.50 | Snow Leopard |
| Snow Leopard | F10874 | 81.50 | Snow Leopard |
| Snow Leopard | F10874 | 81.60 | Snow Leopard |
| Snow Leopard | H21226 | 81.40 | Snow Leopard |
| Snow Leopard | H21226 | 81.50 | Snow Leopard |
| Snow Leopard | H21226 | 81.50 | Snow Leopard |
| Snow Leopard | H21226 | 81.60 | Snow Leopard |
| Snow Leopard | I10579 | 81.40 | Snow Leopard |
| Snow Leopard | I10579 | 81.60 | Snow Leopard |
| Snow Leopard | I10579 | 81.60 | Snow Leopard |
| Snow Leopard | P30654 | 81.40 | Snow Leopard |
| Snow Leopard | P30654 | 81.50 | Snow Leopard |
| Snow Leopard | P30654 | 81.60 | Snow Leopard |
| Snow Leopard | P30654 | 81.40 | Snow Leopard |
| Amur Tiger | B11043 | 74.00 | Tiger |
| Amur Tiger | B11043 | 74.00 | Tiger |
| Amur Tiger | B11043 | 74.10 | Tiger |
| Amur Tiger | B11043 | 74.10 | Tiger |
| Amur Tiger | B11043 | 74.10 | Tiger |
| Amur Tiger | B30631 | 74.00 | Tiger |
| Amur Tiger | B30631 | 74.00 | Tiger |
| Amur Tiger | B30631 | 74.10 | Tiger |
| Amur Tiger | B30631 | 74.10 | Tiger |
| Amur Tiger | B30631 | 73.90 | Tiger |
| Amur Tiger | B30634 | 73.90 | Tiger |
| Amur Tiger | B30634 | 74.00 | Tiger |
| Amur Tiger | B30634 | 74.00 | Tiger |
| Amur Tiger | B30634 | 74.10 | Tiger |
| Amur Tiger | B30634 | 74.10 | Tiger |
| Amur Tiger | C40349 | 74.00 | Tiger |
| Amur Tiger | C40349 | 74.00 | Tiger |
| Amur Tiger | C40349 | 74.10 | Tiger |
| Amur Tiger | C40349 | 74.10 | Tiger |
| Amur Tiger | C40349 | 74.10 | Tiger |
| Amur Tiger | E20760 | 74.10 | Tiger |
| Amur Tiger | E20760 | 74.10 | Tiger |
| Amur Tiger | E20760 | 74.10 | Tiger |
| Amur Tiger | E20760 | 74.10 | Tiger |
| Tiger | E41336 | 74.10 | Tiger |
| Tiger | E41336 | 74.10 | Tiger |
| Tiger | E41336 | 74.10 | Tiger |
| Bengal Tiger | E41366 | 74.10 | Tiger |
| Bengal Tiger | E41366 | 74.10 | Tiger |
| Bengal Tiger | E41366 | 74.10 | Tiger |
| Amur Tiger | G10718 | 74.00 | Tiger |
| Amur Tiger | G10718 | 74.10 | Tiger |
| Amur Tiger | G10718 | 74.10 | Tiger |
| Amur Tiger | H10342 | 74.00 | Tiger |
| Amur Tiger | H10342 | 74.10 | Tiger |
| Amur Tiger | H10342 | 74.10 | Tiger |
| Amur Tiger | H10415 | 74.00 | Tiger |
| Amur Tiger | H10415 | 74.10 | Tiger |
| Amur Tiger | H10415 | 74.20 | Tiger |
| Amur Tiger | H10415 | 74.20 | Tiger |
| Amur Tiger | I30839 | 74.00 | Tiger |
| Amur Tiger | I30839 | 74.10 | Tiger |
| Amur Tiger | I30839 | 74.10 | Tiger |
| Amur Tiger | I41024 | 74.00 | Tiger |
| Amur Tiger | I41024 | 74.10 | Tiger |
| Tiger | J20139 | 74.00 | Tiger |
| Tiger | J20139 Tiger | 74.00 | Tiger |
| Tiger | J20139 Tiger | 74.10 | Tiger |
| Tiger | J20515 | 73.90 | Tiger |
| Sumatran Tiger | J20515 | 74.00 | Tiger |
| Sumatran Tiger | J20515 | 74.00 | Tiger |
| Tiger | K30802 | 74.00 | Tiger |
| Amur Tiger | K30802 | 74.00 | Tiger |
| Amur Tiger | K30802 | 74.00 | Tiger |
| Amur Tiger | K30802 | 74.10 | Tiger |
| Amur Tiger | K40147 | 74.00 | Tiger |
| Amur Tiger | K40147 | 74.00 | Tiger |
| Amur Tiger | K40147 | 74.10 | Tiger |
| Amur Tiger | K40147 | 74.10 | Tiger |
| Tiger | L31074 Tiger | 74.00 | Tiger |
| Tiger | L31074 Tiger | 74.00 | Tiger |
| Tiger | L31074 Tiger | 74.00 | Tiger |
| tiger | L31074 Tiger | 74.10 | Tiger |
| Amur Tiger | N10927 | 73.90 | Tiger |
| Amur Tiger | N10927 | 74.00 | Tiger |
| Amur Tiger | N10927 | 74.10 | Tiger |
| Malayan Tiger | N30772 | 74.10 | Tiger |
| Malayan Tiger | N30772 | 74.10 | Tiger |
| Malayan Tiger | N30772 | 74.10 | Tiger |
| Amur Tiger | I41024 | 74.40 | Inconclusive |
| Bengal Tiger | E41366 | 74.50 | Inconclusive |
| Tiger | E41336 | 74.60 | Inconclusive |
| Amur Tiger | H10342 | 74.70 | Inconclusive |
| Amur Tiger | G10718 | 80.60 | Inconclusive |
| Malayan Tiger | N30772 | 78.30 | Cheetah |

**Table S9**. Individual qPCR melt temperature test results of positive control gBlocks used to determine the expected melt peak temperature for each of the six big cat species, summarized in Table 4.

| **Well #** | **Species** | **Melt Temperature (C)** | **Notes** |
| --- | --- | --- | --- |
| F01 | Jaguar | 82.30 |  |
| F02 | Jaguar | 82.30 |  |
| D01 | Jaguar | 82.40 |  |
| D02 | Jaguar | 82.40 |  |
| D01 | Jaguar | 82.40 |  |
| D02 | Jaguar | 82.40 |  |
| F01 | Jaguar | 82.40 |  |
| F02 | Jaguar | 82.40 |  |
| D05 | Jaguar | 82.50 |  |
| F02 | Jaguar | 82.30 |  |
| F01 | Jaguar | 82.40 |  |
| E05 | Snow Leopard | 81.50 |  |
| G02 | Snow Leopard | 81.40 |  |
| G01 | Snow Leopard | 81.40 |  |
| G02 | Snow Leopard | 81.40 |  |
| G01 | Snow Leopard | 81.50 |  |
| E02 | Snow Leopard | 81.40 |  |
| E01 | Snow Leopard | 81.50 |  |
| E01 | Snow Leopard | 81.60 |  |
| E02 | Snow Leopard | 81.60 |  |
| E02 | Snow Leopard | 81.40 |  |
| E01 | Snow Leopard | 81.50 |  |
| C01 | Lion | 80.90 | (>50,0000 copies) |
| C02 | Lion | 80.90 | (>50,0000 copies) |
| C02 | Lion | 80.90 | (>50,0000 copies) |
| B01 | Lion | 79.00 | (<50,0000 copies) |
| B01 | Lion | 79.10 | (<50,0000 copies) |
| B01 | Lion | 79.10 | (<50,0000 copies) |
| B01 | Lion | 79.30 | (<50,0000 copies) |
| B01 | Lion | 79.30 | (<50,0000 copies) |
| B02 | Lion | 79.10 | (<50,0000 copies) |
| B02 | Lion | 79.10 | (<50,0000 copies) |
| B02 | Lion | 79.10 | (<50,0000 copies) |
| B02 | Lion | 79.20 | (<50,0000 copies) |
| B02 | Lion | 79.30 | (<50,0000 copies) |
| C01 | Lion | 79.00 | (<50,0000 copies) |
| C01 | Lion | 79.10 | (<50,0000 copies) |
| C01 | Lion | 79.00 | (<50,0000 copies) |
| C01 | Lion | 79.10 | (<50,0000 copies) |
| C01 | Lion | 79.20 | (<50,0000 copies) |
| C02 | Lion | 79.10 | (<50,0000 copies) |
| C02 | Lion | 79.00 | (<50,0000 copies) |
| C02 | Lion | 79.10 | (<50,0000 copies) |
| C02 | Lion | 79.20 | (<50,0000 copies) |
| D01 | Leopard | 79.00 |  |
| D01 | Leopard | 79.10 |  |
| D01 | Leopard | 79.10 |  |
| D02 | Leopard | 79.00 |  |
| D02 | Leopard | 79.00 |  |
| D02 | Leopard | 79.10 |  |
| D05 | Leopard | 79.10 |  |
| E01 | Leopard | 79.00 |  |
| E01 | Leopard | 79.10 |  |
| E02 | Leopard | 79.00 |  |
| E02 | Leopard | 79.00 |  |
| F01 | Cheetah | 78.30 |  |
| F02 | Cheetah | 78.30 |  |
| F01 | Cheetah | 78.30 |  |
| F02 | Cheetah | 78.30 |  |
| G01 | Cheetah | 78.30 |  |
| G02 | Cheetah | 78.40 |  |
| G01 | Cheetah | 78.50 |  |
| G01 | Cheetah | 78.30 |  |
| G02 | Cheetah | 78.30 |  |
| A01 | Tiger | 74.00 |  |
| A02 | Tiger | 74.00 |  |
| A01 | Tiger | 74.00 |  |
| A02 | Tiger | 74.00 |  |
| A02 | Tiger | 74.00 |  |
| A01 | Tiger | 74.10 |  |
| A01 | Tiger | 74.10 |  |
| A02 | Tiger | 74.10 |  |
| A01 | Tiger | 73.90 |  |
| A02 | Tiger | 74.00 |  |

**Table S10**. The qPCR Cq values obtained after amplifying tiger bone samples (steeped in alcohol for varying amounts of time before DNA extraction) with the big cat multiplex. No amp = no amplification. NA = not applicable.

| **Steeped alcohol Cq values** | |  |  |  |  |
| --- | --- | --- | --- | --- | --- |
| DNA extraction day | Cq1 | Cq2 | Cq3 | Average Cq | Standard Deviation Cq |
| 36 | No amp | 33.31 | No amp | 33.31 | NA |
| 150 | No amp | 33.34 | 35.03 | 34.185 | 1.20 |
| 294 | 35.57 | 35.23 | 35.19 | 35.33 | 0.03 |

**Table S11**. The Cq values and melt peak temperatures obtained using tiger primers and a serial dilution of the tiger gBlock (Table S2).

| **Tiger gBlock serial dilution Cq values** | | |  |  |  |
| --- | --- | --- | --- | --- | --- |
| gBlock copy number | Cq1 | Cq2 | Cq3 | Average Cq | Standard  Deviation Cq |
| 5 | 31.62 | 32.33 | 31.81 | 31.92 | 0.37 |
| 50 | 26.66 | 26.94 | 26.55 | 26.72 | 0.20 |
| 500 | 23.86 | 23.97 | 23 | 23.61 | 0.53 |
| 5000 | 20 | 20.09 | 19.48 | 19.86 | 0.33 |
| 50000 | 16.2 | 16.82 | 18 | 17.01 | 0.91 |
| 500000 | 13.7 | 13.4 | 13.17 | 13.42 | 0.27 |
| 5000000 | 10.72 | 9.4 | 10.38 | 10.17 | 0.69 |
